# Sporecasting Biogeography Across Scales: Continental Patterns and Local Constraints on Macrofungal Dispersal

**DOI:** 10.1111/mec.70366

**Published:** 2026-05-06

**Authors:** Andrew W. Ratz, A. Elizabeth Arnold, Haley M. Burrill, Marcos V. Caiafa, John Conery, Heather A. Dawson, Carolyn A. Delevich, Jeff M. Diez, Serita D. Frey, D. Jean Lodge, Eivind K. Ronold, Bitty A. Roy, Matthew E. Smith, Jana U'Ren, Andrew W. Wilson, Justin J. Loucks, Jennie Wuest, C. Benton Willis, Peter G. Kennedy

**Affiliations:** ^1^ Department of Plant & Microbial Biology University of Minnesota Saint Paul Minnesota USA; ^2^ University of Arizona Tucson Arizona USA; ^3^ Institute of Ecology and Evolution University of Oregon Eugene Oregon USA; ^4^ Department of Plant Pathology University of Florida Gainesville Florida USA; ^5^ Departamento de Botânica Universidade Federal de Santa Catarina Florianópolis Brazil; ^6^ University of New Hampshire Durham New Hampshire USA; ^7^ University of Georgia Athens Georgia USA; ^8^ Department of Biology University of Oslo Oslo Norway; ^9^ Washington State University Pullman Washington USA; ^10^ Denver Botanic Gardens Denver Colorado USA

**Keywords:** biodiversity, biogeography, functional guild, fungi, spore

## Abstract

Understanding how aerial fungal diversity varies across space, habitat, and disturbance is key to linking local‐scale sporocarp (fruiting body) reproduction with continental‐scale biogeography. In this study, we combined passive spore trap metabarcoding with macrofungal sporocarp records from 31 plots in eight sites across North America to quantify how site, habitat, and fire history shape aerial macrofungal communities. From 823 samples, we recovered 7572 OTUs, nearly half belonging to macrofungi. Macrofungal aerial DNA abundance and OTU richness varied strongly among sites and increased significantly with both temperature and precipitation. Across sites, forest habitats had significantly greater macrofungal aerial DNA abundance than grassland habitats, while conifer forests supported significantly higher OTU richness than oak forests or grasslands. Burn history, likely due to lags in the sampling time following fire, did not significantly impact macrofungal aerial DNA abundance and OTU richness and also had limited effects on community composition. Aerial macrofungal community similarity declined with geographic distance, with geography explaining the most variation (16%). Functional guild composition varied across habitats, shifting from higher ectomycorrhizal prevalence in forest habitats to higher soil saprotroph prevalence in grassland habitats, although wood saprotrophs were the most abundant guild across all habitats. Integration with iNaturalist sporocarp records indicated a mean effective spore trap input range of ~3 km, consistent with strong local dispersal limitation. Together, these findings demonstrate that aerial macrofungal assemblages exhibit pronounced continental‐scale structure yet remain locally heterogeneous, shaped primarily by environmental filtering, functional guild association, and limited dispersal.

## 1 Introduction

Species distributions are shaped by environmental heterogeneity operating at multiple spatial scales (Tilman 1982; Oliver et al. 2010; Chenqi et al. 2022). At continental and global scales, climatic gradients in temperature and precipitation, as well as geographic barriers such as mountain ranges, exert a strong influence over species distributions (Gaston 2000; Weir and Price 2011; Coelho et al. 2023). At finer local scales, other factors (e.g., soil pH, nutrient availability, slope and orientation, host availability, and microclimate) play critical roles in structuring the richness and composition of many ecological communities, particularly those of plants and microbes (Daws et al. 2002; Rousk et al. 2010; Crandall and Gilbert 2017; Jucker et al. 2018; Xie et al. 2025). Within this extensive range of spatial environmental heterogeneity, varying disturbance regimes and processes, such as stochastic dispersal, can further influence patterns of community assembly at both broad and fine scales (Fukami 2004; Dove et al. 2021; Loke and Chrisholm 2023).

Dispersal is a key mechanism by which organisms navigate environmental heterogeneity (Sorensen and Shade 2020; Begon and Townsend 2020). In many ecosystems, dispersal is directly observed via the movement of animals or macroscopic propagules such as seeds. However, despite being among the most diverse and ecologically influential groups of organisms on Earth, the dispersal of fungi remains less understood (Peay et al. 2016; Baldrian et al. 2022; Cuprewich et al. 2025), in part because microorganisms were long assumed to be ubiquitous, and only recently have studies begun to reveal their biogeographical patterns and dispersal processes (Martiny et al. 2006; Golan and Pringle 2017). The microscopic propagules used by fungi to disperse (spores) represent both the reproductive output of fungal populations and the basis for future community assembly (Womack et al. 2015; Aguilar‐Trigueros et al. 2019), yet their sources, movement, and ecological outcomes are often difficult to trace (Chaudhary et al. 2022).

Recent advances in sample capture and high‐throughput DNA sequencing have substantially advanced knowledge about fungal spore dispersal. At the global scale, Abrego et al. (2024) revealed that airborne fungal communities are strongly structured by latitude, season, and geographical distance, showing clear biogeographic patterns. At the same time, that study, as well as surveys at smaller spatial scales, indicate that stochasticity plays a significant role in community composition, even at the scale of meters (Peay et al. 2012; Smith and Peay 2021). At ecoregional and landscape scales, studies have demonstrated that airborne fungal assemblages are shaped by climatic and habitat gradients, local land use, geographic barriers, and wind patterns (Peay and Bruns 2014; Branco et al. 2015; Crandall and Gilbert 2017; Pellitier et al. 2025; Wagner et al. 2022; Naranjo‐Orrico et al. 2025). Still, not all places follow the same patterns: in isolated tropical locations, such as Hawai‘i, airborne fungal communities remained remarkably stable over more than a decade, suggesting limited environmental influence and high temporal stability (Tipton et al. 2019). Taken together, these studies highlight that, while airborne fungal communities often reflect predictable biogeographic patterning and climatic structuring, their dynamics can also be shaped by unique local contexts.

Connecting spore dispersal patterns to the organisms that produce them remains a challenge, especially given the underrepresentation of fungal biodiversity in sporocarp‐based surveys compared to methods assessing DNA found in the environment (Baptista et al. 2015; Runnel et al. 2024). Focusing on macrofungi, i.e., species that produce conspicuous and often ephemeral sporocarps, in these broader DNA‐based methods may offer a valuable perspective on bridging this gap. Macrofungal fruiting events are directly observable, spatially localised, and temporally constrained, thus providing tangible links between airborne propagule pools and habitat‐specific ecological processes (Peay et al. 2012; Borgmann‐Winter et al. 2023). Importantly, macrofungal observations can thus serve as ‘ecological benchmarks’ in the interpretation of DNA‐based datasets (Peay et al. 2007; Vasco‐Palacios et al. 2018). Efforts to connect airborne fungal spore communities to sporocarp production have begun to shed more light on these dynamics (Castaño et al. 2017), but such work has largely been restricted to specific habitats or fungal guilds (e.g., Abrego et al. 2018).

In this study, we implemented a replicated sampling design spanning multiple ecoregions, habitat types, and fire histories in North America to test how aerial macrofungal community composition responded to abiotic and biotic variation at both continental and local scales. We predicted that, at the broad continental scale, aerial macrofungal community abundance and structure would be primarily driven by site identity, with additional influence from precipitation and, to a lesser degree, temperature, as seen in previous studies of fungal biogeography (Tedersoo et al. 2014; Abrego et al. 2024). By sampling common habitat types, e.g., forests and grasslands, across most of our study sites, we also sought to assess the effects of habitat‐driven patterns relative to those of broader bioclimatic‐ and distance‐based effects. We expected the targeted habitats to vary significantly in aerial macrofungal abundance and community structure, with forests supporting a greater diversity and abundance, as well as a higher compositional variability, than grasslands. This expectation was based on the elevated stature of many macrofungi (e.g., polypore fungi fruiting on trees (Abrego et al. 2018)) and the higher representation of macrofungal sporocarps in these habitats due to ectomycorrhizal sporocarp fruiting (Van Nuland et al. 2025) as well as the higher abundance of aboveground biomass such as downed wood, which serves as both a fungal habitat and can increase preferred microclimatic conditions (Wijas et al. 2024). We further incorporated paired burned and unburned plots nested within each habitat type to assess how disturbance history modifies aerial macrofungal communities. Fire is well known to alter vegetation, nutrient availability, and microhabitat structure (Davies 2013), and previous studies show that it can often reduce fungal richness and favour pyrophilous taxa, distinctly altering successional trajectories (Baar et al. 1999; Hughes et al. 2020; Brown et al. 2021). Therefore, we predicted that recently burned locations would exhibit lower overall spore richness and altered community composition relative to unburned locations within the same habitat and ecoregion. Finally, because landscape heterogeneity and small‐scale environmental variation can create spatially patchy spore dispersal, we expected that the ‘effective input range’ of spores from sporocarps into airborne pools would be limited, likely less than 5–10 km (Lodge 1985; Peay et al. 2012; Abrego et al. 2018). To test this final hypothesis, we utilised macrofungal sporocarp occurrence records deposited in the iNaturalist database, collected by both our research team as well as public community members near each site. Collectively, by integrating multi‐scale spatial coverage, habitat replication, fire history, and sporocarp records into a unified sampling framework across a range of geographically distant North American sites, we aimed to clarify the relative roles of distance, environmental filtering, and disturbance in shaping airborne macrofungal biodiversity at both local and continental scales.

## 2 Materials and Methods

### 2.1 Study Sites

**FIGURE 1:**
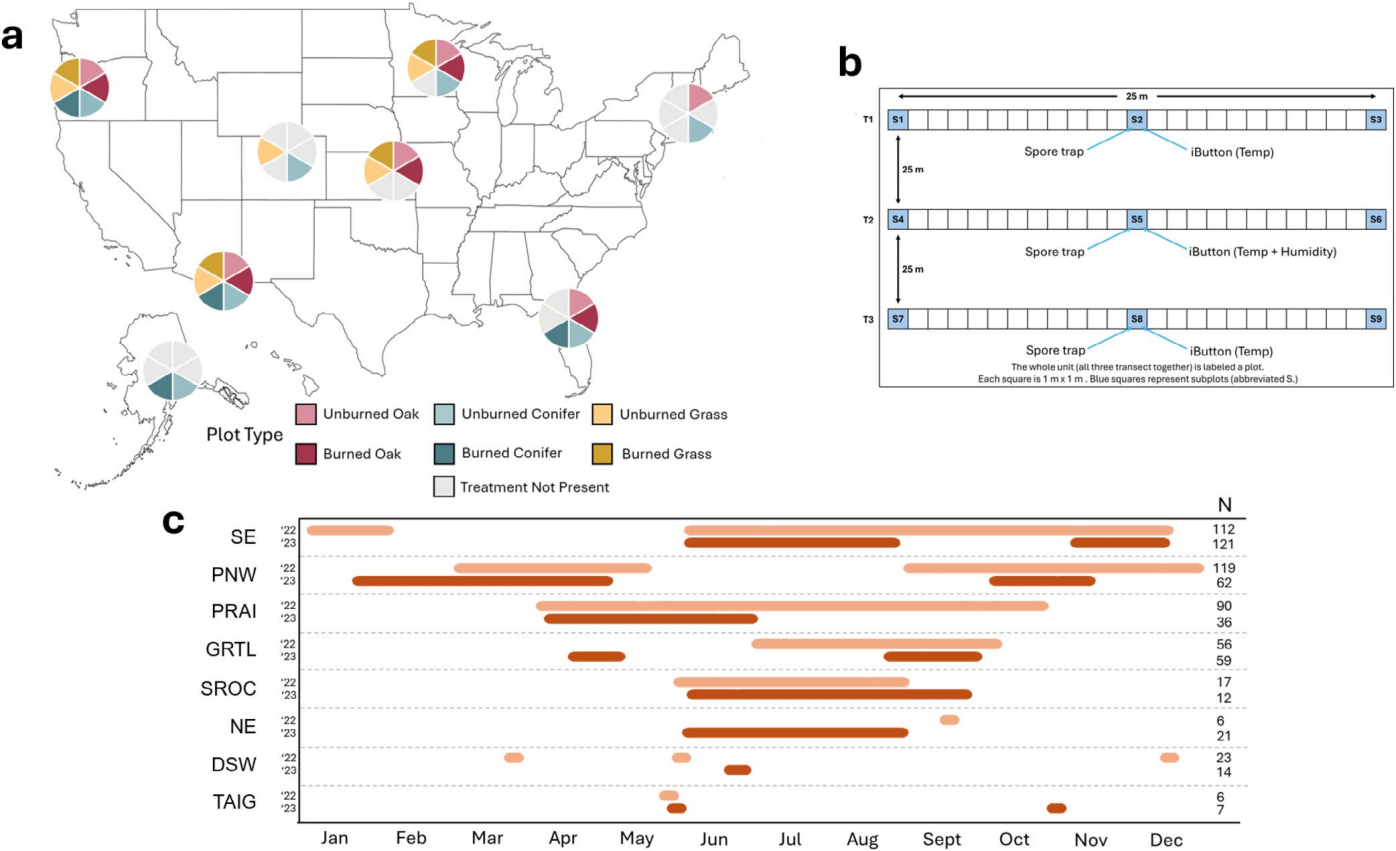
Study sampling regime across sites, treatments, and time. Map of the United States (a) showing the sampling locations and the high, but somewhat unbalanced, treatment replication across sites. The sampling design within each habitat and burn treatment (b) was similar across all sites. Spore traps were always located in the middle of sampling transects along with climate sensors. The sampling window (c) across 2022 (light orange) and 2023 (dark orange) was inconsistent across sites due to logistical constraints such as snowfall, insufficient personnel, or lack of access to sites.

Our sampling sites consisted of 31 plots distributed across 8 National Ecological Observatory Network (NEON) and NSF Long Term Ecological Research (LTER) network sites (Figure 1). Sites were selected to capture a broad range of climatic and geographic conditions spanning 8 major ecoregions of the United States (Table S1). Briefly, the sites spanned a wide range of precipitation (MAP: 25–133 mm), temperature (MAT: −1.2°C–16°C), elevation (21–3500 m), continental location (represented states include Alaska, Arizona, Colorado, Florida, Kansas, Massachusetts, Minnesota, and Oregon), and vegetational composition. Due to a lack of replication within the eight ecoregions, we apply the names for each ecoregion to provide ecological context about their location but emphasise that our inferences are limited to only these eight sites rather than the entire ecoregion from which they were sampled. Plots represented combinations of three habitat types—grasslands, Pinaceae‐dominated conifer forests, and oak‐dominated deciduous forests—with varied fire histories: recently burned (typically within the past 5 years) and unburned for multiple years to decades. Due to logistical constraints, not all habitat and fire regime combinations (hereafter referred to as treatments) were available for sampling at every site (Figure 1a).

### 2.2 Spore Sampling

Within each site, plots representing targeted habitats and burn histories were located 50 m to 60 km apart depending on local conditions/availability. Each plot contained 3 parallel 25 m by 1 m transects, spaced 25 m apart (Figure 1b). Spore traps were located at the center location of each transect and were constructed using a passive spore trap design modified by Borgmann‐Winter et al. (2023) from Peay et al. (2012). Each spore trap consisted of a 10‐cm diameter funnel, fitted with a 500 μm mesh (U‐CMN‐500, Component Supply, Sparta, Tennessee, USA) to exclude large debris, placed in a 1 L plastic bottle containing 200 mL of 100% ethanol to prevent in situ microbial growth during the collection period. To further minimise contamination from splash and surface disturbances, a 36 × 36 cm piece of landscape fabric was placed under and around each trap to further reduce soil contamination during rain events.

Spore traps were collected weekly to tri‐monthly over 2 years, with intervals varying across sites (median collection time = 27 days, range = 5–90 days) and were emptied as needed. If there was little rainfall, the containers were collected less often (e.g., Bonanza Creek, AK in the Taiga ecoregion) and where there was more rainfall (e.g., Ordway‐Swisher, FL in the Southeast ecoregion) they were collected more often. More detailed information regarding the targeting of collection timeframes can be found in the Supporting Information. Upon collection, spore trap contents (e.g., ethanol and rainwater) were refrigerated within 48 h and then vacuum‐suctioned onto 1–2 μm cotton filter paper (Sartorius FT3211090, Thermo Fisher Scientific, Waltham, Massachusetts, USA). The filters were labelled and frozen in sterile plastic bags at −20°C until shipment to the University of Minnesota at the end of each sampling season for processing.

### 2.3 Molecular Quantification and Identification

Upon thaw, the surface of each filter was scraped using sterilised scalpels and all contents were transferred into 2 mL Qiagen PowerBead tubes (Qiagen, Hilden, Germany). If multiple filters were used due to excess particulate matter (dust) in a sample, a proportional amount was scraped from each filter to ensure that the total particulate matter sequenced represented the equivalent of a single filter. DNA extraction followed the Qiagen DNeasy PowerSoil Pro Kit protocol, with a final elution volume of 100 μL.

To quantify total fungal DNA, we used quantitative PCR (qPCR), which is an established proxy for spore DNA abundance in aerial communities that has been shown to have strong but varied correlation with spore counts depending on the taxa of focus (Ovaskainen et al. 2020; Wang et al. 2025; Pogner et al. 2025). Specific qPCR amplification methods and conditions can be located within the Supporting Information. We partitioned total fungal DNA abundance to specifically estimate macrofungal DNA abundance by calculating the relative abundance of macrofungal OTUs within each sample from the high throughput sequencing data (detailed below) and scaling the total fungal DNA by this relative proportion, as in Mielke et al. (2025). While we recognise that this approach assumes a correlation between sequence read proportions and aerial biomass, we believe this proportionalisation provides a more representative estimate of aerial macrofungal DNA abundance than using the abundance results for all fungal DNA, which includes many non‐target microfungal taxa. To assess the composition of aerial macrofungal communities, the ITS1 region was targeted with the same primer pair also containing Illumina adapters (Integrated DNA Technologies, Coralville, Iowa, USA) following the protocols outlined in Gohl et al. (2016). PCR amplification was performed using 30 cycles with an annealing temperature of 55°C. A second round of amplification was conducted to add unique sample barcodes for high‐throughput sequencing. The synthetic mock community of Palmer et al. (2018), as well as multiple DNA extraction controls consisting of no spore material, were included for quality control processing. Barcoded amplicons were purified and normalised using SequalPrep kits (Invitrogen, St. Louis, Missouri, USA). Final libraries were then sequenced at the University of Minnesota Genomics Center on a single Illumina NextSeq 2000 platform P2 run, generating 2 × 300 bp paired‐end reads.

Raw sequences were initially processed using cutadapt (Martin 2011) to trim primers and sequencing adapters from all reads, keeping only sequences where both the forward‐tag and reverse‐tag matched the mapping files. Quality filtering, denoising, and merging were performed using DADA2 (Callahan et al. 2016), with recommended parameters for ITS (maxN = 0, maxEE = c(2, 2), truncQ = 2, minLen = 50) to generate Amplicon Sequence Variants (ASVs). To account for intraspecific variation in the ITS region, which can cause DADA2 to incorrectly partition sequences from the same operational species into separate ASVs (Estensmo et al. 2021), we clustered ASVs into Operational Taxonomic Units (OTUs), with specific methods being available in the Supporting Information. To minimise over splitting of OTUs, the mumu algorithm was applied (Mahé 2021; https://github.com/frederic‐mahe/mumu) on the resulting OTU table. Taxonomic assignment and bootstrap calculation for all taxonomic ranks were performed using the SINTAX classifier (Edgar 2016) with the UNITE database (ITS version 10.0, released on 04.04.2024 including all eukaryotes, Abarenkov et al. 2024). Before assigning taxonomy, all the taxon headers in the UNITE FASTA file that included ‘unidentified’ were removed using a custom script. Macrofungal OTUs with a genus‐level bootstrap confidence of ≥ 80% were assigned a primary lifestyle classification using the FungalTraits database (Põlme et al. 2020). All of the FASTQ files were deposited in the National Center for Biotechnology Information (NCBI) Short Read Archive (SRA) under BioProject number PRJNA1291903.

The initial OTU data table included 13,126 fungal OTUs across 823 samples, with a total read count of 12,950,425 sequences. The negative control contained 1928 non‐zero reads, of which 1855 were identified as a known skin dandruff contaminant (*Malassezia restricta*). All non‐zero OTUs detected in the negative control were summed and subtracted from each sample in the dataset as in Nguyen et al. (2015). To correct for tag switching across samples (Carlsen et al. 2012), the positive control was assessed for unexpected OTUs, and it was determined that all OTUs with read counts below 15 were considered suspect and therefore removed. Given the high variation in sequencing depth across samples (ranging from 0 to 112,725 reads; mean = 15,000 reads; SD = 12,700 reads), OTU counts were rarefied to 2063 reads per sample. Good's coverage index was calculated for each sample and grouped by site to detect potentially uncaptured richness across sites. The median coverage score for each site was above 0.95, which was deemed acceptable for further analysis. Macrofungal OTUs were defined as non‐lichenised fungi known to produce conspicuous sporocarps, including, but not limited to, agaricoid, secotioid, clavarioid, gasteroid, and resupinate growth forms. These definitions were applied using an expert‐curated list of taxa (Table S3).

### 2.4 Environmental Variation

Plot‐specific temperature and relative humidity were recorded hourly at the center of each plot throughout spore trap deployment using Maxim Integrated iButton sensors (Maxim Integrates, San Jose, CA, USA) (Figure 1b). All iButton sensors were mounted 30 cm above the soil surface on labelled stakes within protective casings to shield them from direct exposure to sunlight and precipitation. Daily temperature, precipitation, and relative humidity data, which were then averaged into mean monthly temperature and mean monthly precipitation during the sampling timeframe, were obtained from the National Ecological Observatory Network (NEON) database for corresponding sampling periods. Data were sourced from local airport weather stations or Long‐Term Ecological Research (LTER) site weather stations for sites lacking NEON precipitation data, including Cedar Creek in the Great Lakes ecoregion, and Mt. Pisgah and H.J. Andrews in the Pacific Northwest ecoregion. Long‐term historical temperature and precipitation averages from 1991 to 2020 were also acquired from the PRISM Climate Group database and used as mean annual temperature and mean annual precipitation metrics for each study site.

### 2.5 Sporocarp Surveys

Macrofungal sporocarp surveys were conducted at least twice annually per site using an intentional meandering method within each plot outlined by Mueller et al. (2004). Trained specialists ran forays with the help of public community members at each site, identifying and collecting visibly distinctive sporocarps. The observation date and location of each collection was recorded in the iNaturalist database, along with multiple photos to facilitate verifiable identifications. DNA was extracted following the methods of Caiafa et al. (2025) and the specimens were then dried for storage in fungaria. In addition to these research‐team surveys, community‐contributed iNaturalist sporocarp records were retrieved for each site during the sampling periods in 2022 and 2023 to provide a broader source of macrofungal occurrence data. All observations identified to genus were included within a 30 km radius from the spore trap locations, based on the spatial range results of Grinn‐Gofroń et al. (2020) and Oteros et al. (2015). To maximise sample size, particularly in less frequently surveyed areas, taxonomic data from iNaturalist datasets were analysed at the genus level. While we recognise that a genus‐level comparison may overestimate community overlap between sporocarp observation and molecular data, this taxonomic level represents the majority of records in both datasets while conserving some level of taxonomic discrimination and largely matches the level at which functional annotations could be assigned (Nguyen et al. 2015).

### 2.6 Statistical Analyses

All statistical analyses were conducted in RStudio (R version 4.3.3, RStudio release September 2024). Species richness and DNA abundance were compared among groups using Kruskal–Wallis rank‐sum tests, with post hoc comparisons, when group size exceeded three and compared using Wilcox tests in small groups due to the non‐parametric nature of the data. Climate and environmental effects on aerial macrofungal OTU richness and abundance were analysed using a series of candidate mixed‐effects models. Given the nested nature of study design, along with the high level of temporal pseudo‐replication possibly inflating *p*‐values due to repeated collection, we included subplot within site as nested random factors (1| site/subplot). We then tested three primary models for each response variable; broad climatic drivers of Mean Annual Temperature (MAT) and Precipitation (MAP), more immediate monthly climate factors of Mean Monthly Temperature (MMT) and Precipitation (MMP), and finally a combined model that allowed for correlation across scales and interaction between the terms. The best‐performing models were selected based on both AIC values and parsimony. Categorical variables were then added to the most parsimonious model to investigate the effect of season, year of collection, and elevation on aerial macrofungal OTU richness and DNA abundance. To assess the variance explained by different predictors, variance partitioning was performed based on Bray–Curtis dissimilarity using the varpart() function in the vegan package (Oksanen et al. 2025). Predictor variables were grouped into categories including geography (latitude, longitude, elevation), climate (monthly and annual precipitation and temperature, later separated into two groups), and time (season and year).

Community composition analyses were also based on Bray–Curtis pairwise dissimilarities. These dissimilarities were compared against haversine geographic distances and further explored with non‐metric multidimensional scaling (NMDS). Group‐level differences were tested using PERMANOVA and homogeneity of dispersion tests using the adonis() and betadisper() functions respectively within the vegan package. When habitat type or burn history were analysed as drivers of community composition, the permutations function within adonis2 was used to ensure any effect seen was based on the tested variable and not due to the incomplete and nested nature of the data. To estimate effective spore input range, breakpoint analyses were conducted using a nonlinear least squares (nls) model fit, with additional separate linear models fit to the exponential curve to determine the point at which the decay model changes slope using the segmented() function in the segmented package (Muggeo 2025; version 2.1‐4). Species accumulation curves were fitted with the Michaelis–Menten model, which estimates both the asymptotic species richness (“a,” the expected richness under infinite sampling effort) and the effective search area (“b,” the distance at which half of the maximum expected richness can be observed).

## 3 Results

**FIGURE 2:**
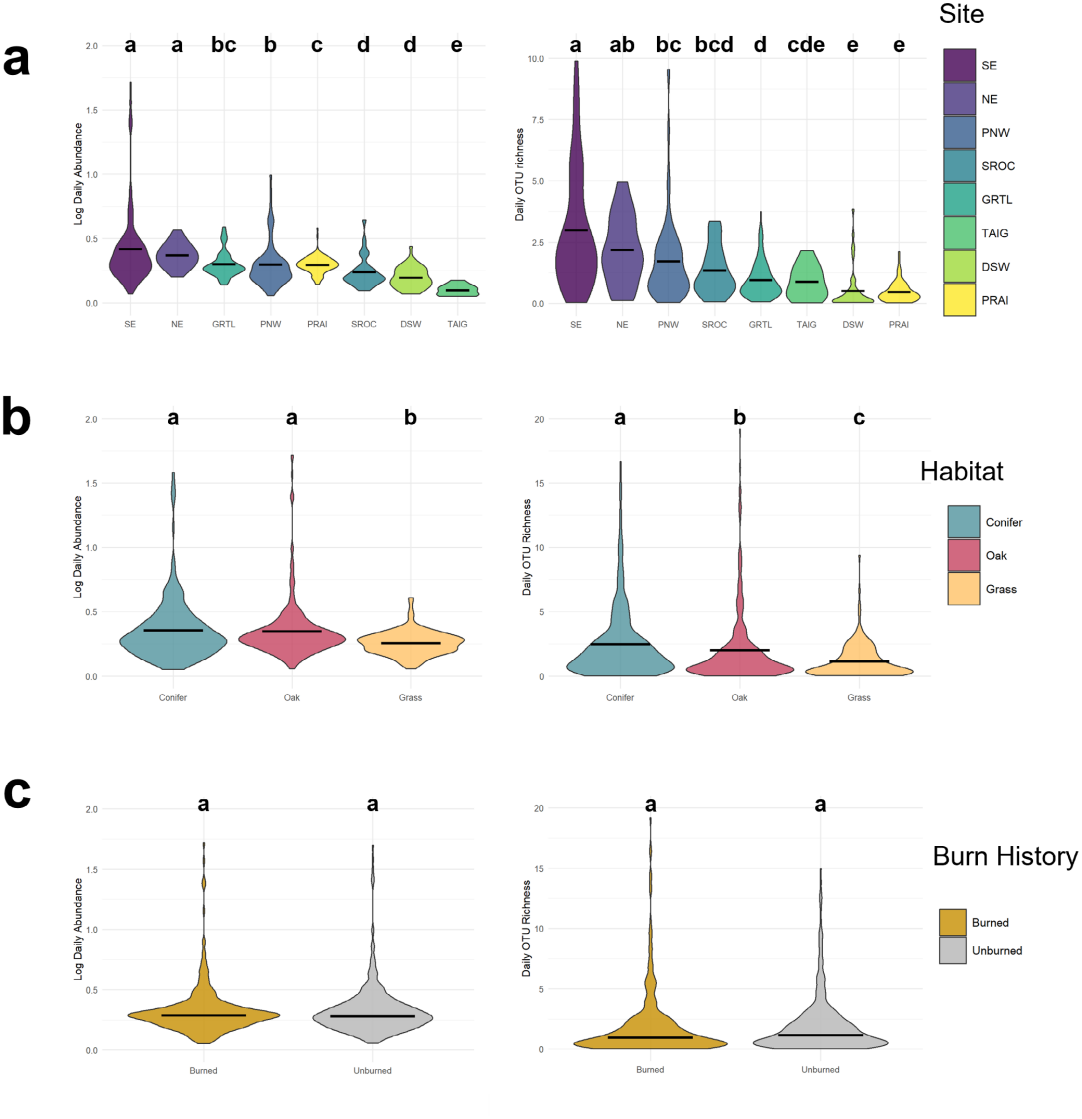
Site and treatment effects on daily aerial macrofungal DNA abundance and OTU richness. Site ecoregion abbreviations are Southeast (SE), Northeast (NE), Pacific Northwest (PNW), Southern Rockies (SROC), Great Lakes (GRTL), Taiga (TAIG), Desert Southwest (DSW), and Prairie (PRAI). Site (a) had an overall significant impact on the daily macrofungal DNA abundance (left; *Χ* ^2^(7) = 139.0, *p* < 0.001) and OTU richness (right; *Χ* ^2^(7) = 275.6, *p* < 0.001). Habitat (b) was a significant driver of and daily macrofungal DNA abundance (left; *Χ* ^2^(2) = 6.70) and OTU richness (right; *Χ* ^2^(2) = 40.78, *p* < 0.001). Burn history (c) had no significant effect on daily macrofungal DNA abundance (left; *p* = 0.91), though OTU richness showed a marginal non‐significant response (right; *p* = 0.08).

From the 7572 fungal OTUs that passed quality control, 3114 (41%) were classified as macrofungi, with 2119 of the macrofungal OTUs (68%) having genus‐level identifications above our 80% bootstrap cutoff. Across the eight sites, 2251 (72%) of the macrofungal OTUs were detected in only one site and 1112 (36%) OTUs were detected in a single sample. Aerial macrofungal DNA abundance (as measured using quantitative PCR) varied considerably across sites, with highest total abundance at the Northeast region site (10565000 copies) and lowest total abundance at the Southern Rockies region site (52100 copies). When correcting for the number of days that spore traps were deployed, mean daily DNA abundances were also highest at the Northeast region site (10,200 copies/filter/day) and lowest at the Southern Rockies region site (98 copies/filter/day), with a highly significant overall difference in mean abundances across ecoregions based on a Kruskal–Wallis test (*Χ* ^2^ (7) = 40.78, *p* < 0.001) (Figure 2). Within sites, habitat had a significant effect on daily DNA macrofungal abundance in the Pacific Northwest region site (*Χ* ^2^(2) = 6.70, *p* = 0.035), but the history of recent fire had no significantly detected effect on daily abundance in any site (*W* = 3999, *p* > 0.34 at the Pacific Northwest region site at minimum, Figure S1b).

The daily observed OTU richness of the aerial macrofungal communities varied significantly across sites (Kruskal–Wallis rank sum test (*p* < 0.001)), with the Southeast ecoregion site having the highest OTU richness overall (1386) and the Taiga ecoregion site having the lowest (332). When averaged across sites, there were also significant differences in daily OTU richness among habitats (*p* < 0.001), with higher OTU richness on average in conifer‐dominated forests than in oak‐dominated forests, which had higher OTU richness than grasslands (Figure 2). Aerial macrofungal OTU richness patterns within sites containing all three habitats, however, were more variable, with some having equivalent richness in forests and grasslands and the Pacific Northwest region site having higher macrofungal OTU richness in grassland samples than those of oak‐dominated forests (Figure S1c). The presence of recent fire consistently had no significant detected effect on aerial macrofungal OTU richness across nor within sites (*p* = 0.26 at minimum in the Great Lakes region site, Figure S1c).

**FIGURE 3:**
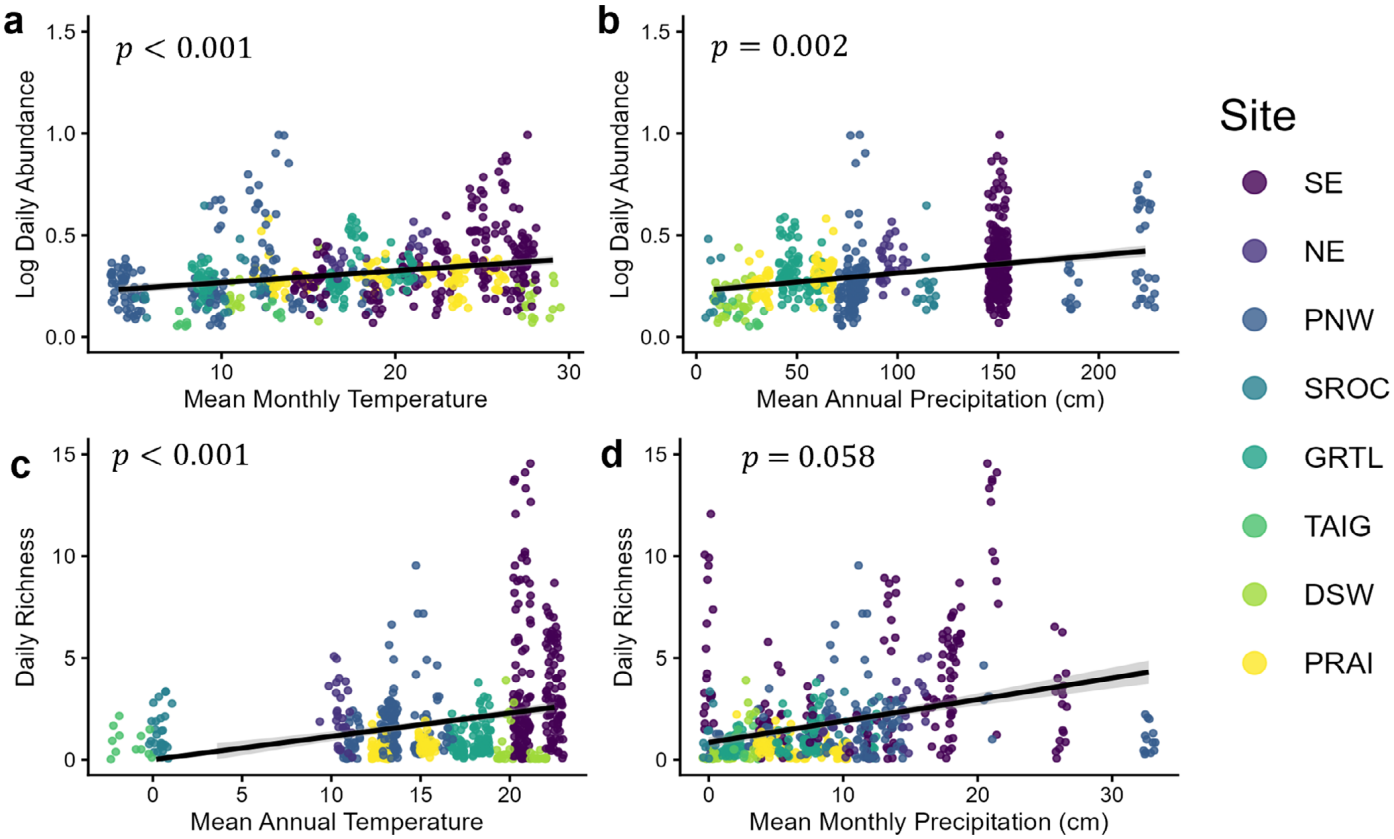
Annual and monthly climate effects on daily aerial macrofungal DNA abundance and OTU richness based on linear mixed modelling. Daily aerial macrofungal DNA abundance was most significantly related to Mean Monthly Temperature (*t*_492.3_ = 4.85, SE = 0.01) and Mean Annual Precipitation (*t*_208.0_ = 2.79, SE = 0.01). Daily aerial macrofungal OTU richness was most strongly related to the latitudinal climatic trends of Mean Annual Temperature (*t*_112.5_ = 5.11, SE = 0.23) and Mean Monthly Precipitation (*t*_650.8_ = 2.20, SE = 0.08). Reported *p*‐values are based on full climatic models including these metrics along with season, elevation, and year of collection. Points are lightly jittered to show the density of measurements and coloured by the Site of spore collection.

Across sites, aerial macrofungal DNA abundance and OTU richness were both significantly linked with climatic variation but were best explained by different climatic measures (Figure 3). Daily macrofungal DNA abundance increased with mean monthly temperature (*t*_326.1_ = 3.11, *p* = 0.002), mean annual precipitation (*t*_21.1_ = 2.92, *p* = 0.008), and varied significantly with season of collection (*t*_666.97_ = 3.56, *p* < 0.001; late season > early season). Fixed effects accounted for 21.2% of the total variance, with nested random effects (subplot within site) increasing the total explained variance to 27.4% (Marginal *R* ^2^ = 0.212; Conditional *R* ^2^ = 0.274). Similarly, daily macrofungal OTU richness increased with mean annual temperature (*t*_81.1_ = 5.34, *p* < 0.001) and mean monthly precipitation (*t*_655.1_ = 2.67, *p* = 0.008). While overall OTU richness was higher in 2023, the year of collection had a negative effect after correcting for climatic covariates (*t*_569.5_ = −2.77, *p* = 0.006). Across all sites, elevation was also positively correlated with macrofungal OTU richness (*t*_86.5_ = 2.12, *p* = 0.037), but this result is likely due to the localised habitat effects within specific sites as described below. Fixed effects explained 24.1% of total variance, while the nested random effects increased the total explained variance to 88.1%, highlighting the role of site in structuring macrofungal OTU richness (Marginal *R* ^2^ = 0.241; Conditional *R* ^2^ = 0.881).

Higher elevation plots, specifically all those found in the Southern Rockies region site and the conifer‐dominated forests in the Desert Southwest region site, had significantly lower macrofungal DNA abundance (*t*_743_ = −7.522, SE = 0.005, *p* < 0.001) and OTU richness (*t*_743_ = −11.69, SE = 0.077, *p* < 0.001) than samples collected in lower elevation sites. When assessed within sites, responses to variation in temperature and precipitation were less consistent. Most sites showed no significant associations between aerial macrofungal DNA abundance and OTU richness with temperature and precipitation. However, the Great Lakes ecoregion site exhibited a positive correlation between abundance and monthly precipitation (*t*_90.5_ = 5.54, *p* < 0.007), while the Southeast and Northeast ecoregion sites showed a positive correlation between richness and monthly temperature (*r* = 0.55, *t*_198_ = 2.12, *p* < 0.016 and *r* = 0.23, *t*_27_ = −2.83, *p* = 0.009, respectively). In contrast, the Prairie ecoregion site showed negative correlations between richness and temperature (*r* = −0.56, *t*_122_ = −2.28, *p* = 0.024) along with a negative correlation between DNA abundance and precipitation (*r* = −0.08, *t*_119_ = −3.88, *p* < 0.001).

**FIGURE 4:**
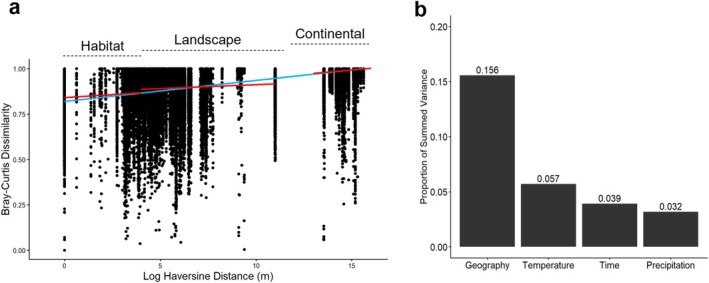
Ecological drivers of aerial macrofungal beta diversity. (a) Relationship between community dissimilarity (Bray–Curtis) and geographic distance (log Haversine distance) between all sampling locations. (b) Proportion of variance in macrofungal OTU composition explained by Geography (site, distance, and elevation), Temperature (MAT and MMT), Precipitation (MAP and MMP), and Time (season and year). Combined, these variables explained a total of 19% of the total community variation, with all overlapping variance between groups reported as a single additive percentage.

Aerial macrofungal community similarity exhibited a significant, but highly variable, distance‐decay trend across all three noted spatial scales (Figure 4). Geographic variables (latitude, longitude, site, and altitude) provided the best explanation of variation in aerial macrofungal community composition, accounting for ~16% of the total variation when including interactive effects with other variables (*R* ^2^_adj_ = 0.16). Temperature explained a total of ~6% of the total variance when including interactions (*R* ^2^_adj_ = 0.06), followed by year and season (~4%, *R* ^2^_adj_ = 0.04) and finally precipitation (~3%, *R* ^2^_adj_ = 0.03). Notably, ~81% of the total variance could not be attributed to measured variables. Aerial macrofungal community composition differed significantly among sites (PERMANOVA: *R* ^2^ = 0.146, *p* < 0.001), with both centroid positions and dispersions showing strong variation (Betadisper: *F*_7,727_ = 27.9, *p* < 0.001). The Desert Southwest region site exhibited the greatest compositional dispersion, consistent with its large distance and elevation differences among sampling plots along with lower overall spore sampling effort.

**FIGURE 5:**
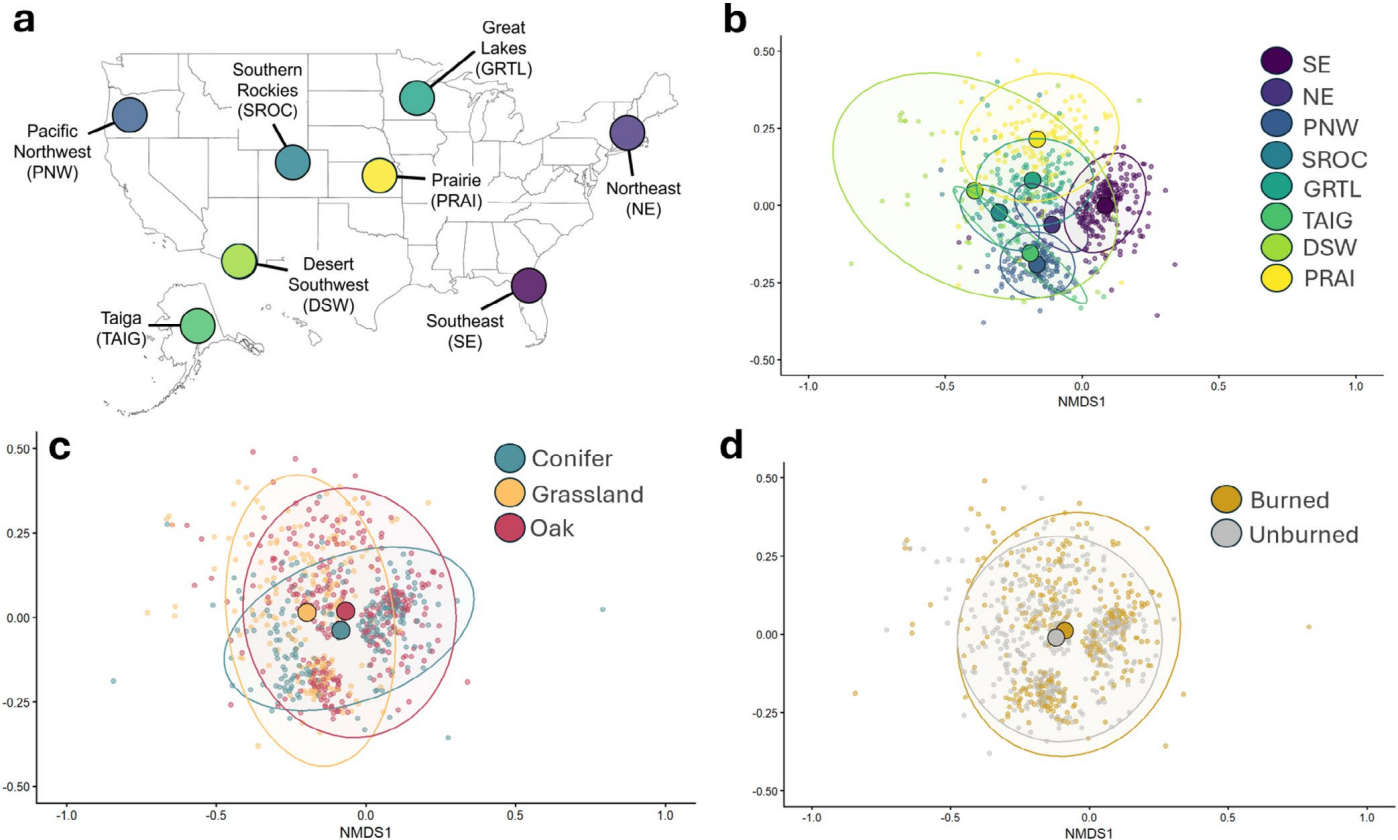
Compositional differences in aerial macrofungal communities across the 8 ecoregion sites (a) based on Bray–Curtis dissimilarity by site (b), habitat (c), and burn history (d). Non‐Metric Multidimensional Scaling Plot Stress = 0.127.

Across sites, PERMANOVA results using restricted permutations to account for nesting showed that site was the primary driver of community composition (*R* ^2^ = 0.135, *p* = 0.010). Habitat did not significantly influence aerial composition across the continental scale (*R* ^2^ = 0.011, *p* = 0.097), whereas burn history had a small but consistent influence on overall aerial macrofungal communities (*R* ^2^ = 0.003, *p* < 0.001; Figure 5). Within sites (excluding the Southern Rockies and Northeast region sites), habitat significantly structured composition (*R* ^2^ range: 0.02–0.10, *p*_range_ < 0.001–0.002), with all habitats differing from one another (*p*_max_ = 0.024; Figure S2). The effect of habitat was weakest in the Desert Southwest region site, while pairwise habitat differences were weakest in the Great Lakes region site. Recent fire history had a significant, but low impact on composition within the Pacific Northwest, Great Lakes, Prairie, and Southeast region sites (*R* ^2^ range: 0.010–0.016, *p*_max_ = 0.024 in Pacific Northwest region site).

**FIGURE 6:**
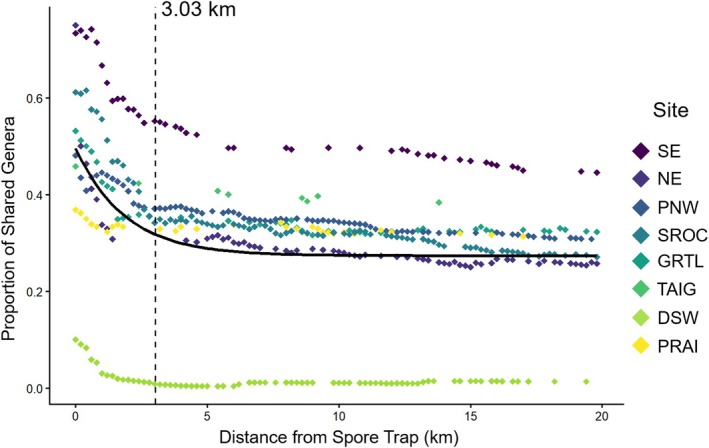
Proportion of genera shared between macrofungi encountered in the spore trap molecular dataset and iNaturalist sporocarp observations during the sampling period, as a function of distance from the spore trap. Each site is plotted in a different colour, with points denoting the cumulative proportion of shared genera up to that distance in 200 m breaks. The dotted line shows the breakpoint as described using segmented linear models (*p* < 0.022) and describes the mean range at which iNaturalist sporocarp community significantly overlaps with collected spores using spore traps (3.03 km).

iNaturalist sporocarp records across the sites filtered to spore sampling times totaled 14,121 observations representing 267 genera. The Pacific Northwest region site had the highest number of observations (6337) and the Desert Southwest region site the lowest (36), with a mean of 1765 observations from 106 genera per site. The proportion of shared genera by distance curves varied across sites, with an average effective sampling distance of 3.03 ± 0.89 km (Figure 6). Some sites, particularly those with low numbers of sporocarp records (e.g., DSW, PRAI), had low initial proportions and quickly levelled out, while others with high observation numbers (e.g., SE, GRTL, and SROC), had high initial proportions with a steep curve, but still came to an asymptote by ~5 km (Figure S4).

**FIGURE 7:**
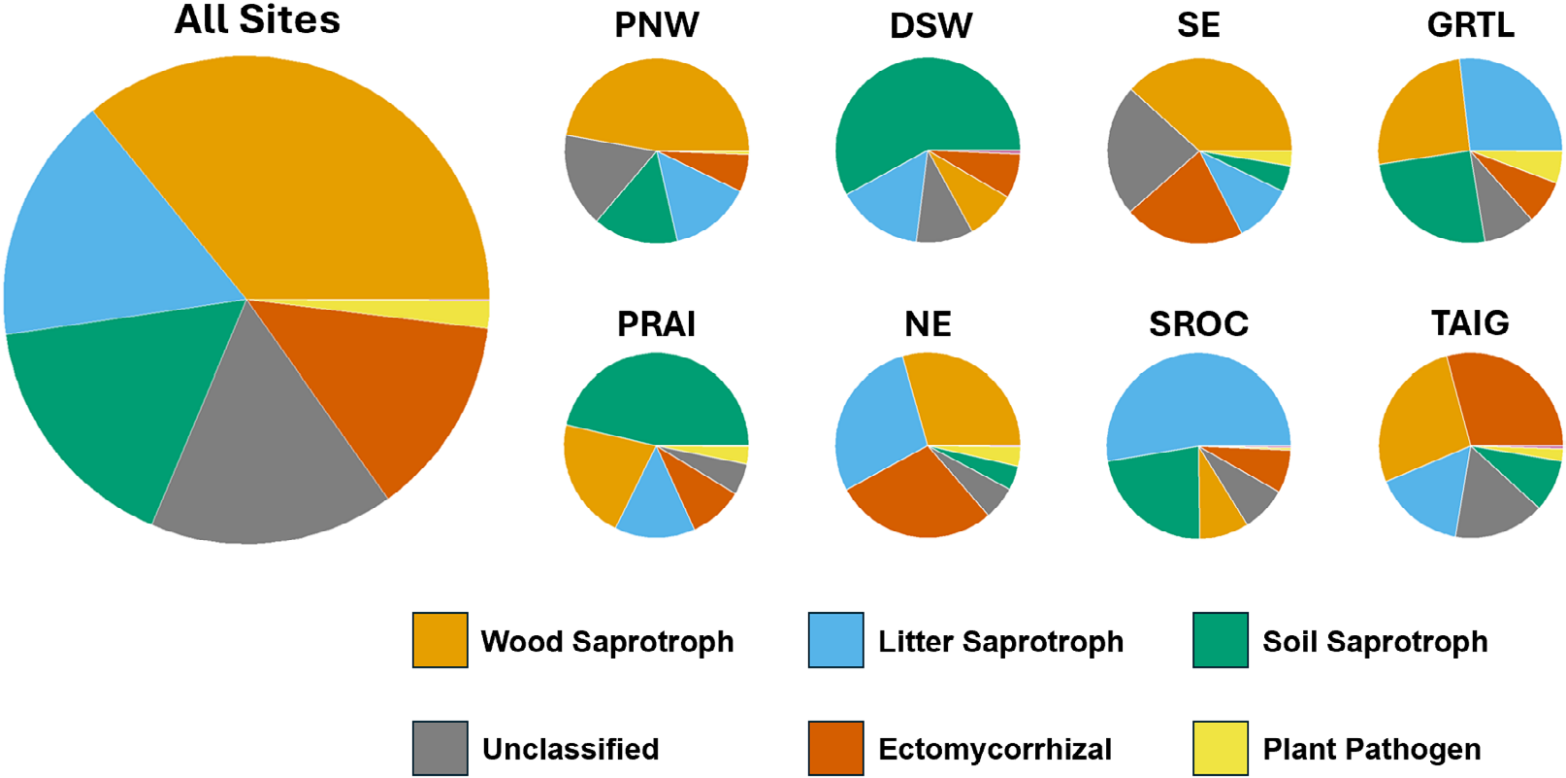
Aerial macrofungal functional guild distribution among and within sites. Fungal taxa were identified to genus (denoted as unclassified if the genus level confidence was < 80% based on SINTAX assignment of taxonomy) and assigned to a functional guild using the FungalTraits database.

Functional guild composition of the aerial macrofungal communities also varied across sites (Figure 7). The most abundant guilds were wood saprotrophs (36.0% of all OTUs), litter and soil saprotrophs (each 16.1%), ectomycorrhizal fungi (13.5%), and plant pathogens (2%). A minor fraction of the remaining taxa represented other lifestyles, including dung saprotrophs, lichen‐parasitic fungi, mycoparasites, and arthropod‐associated fungi as well as some macrofungi identified to genus with no assigned primary guild. Wood saprotrophs were particularly abundant across all sites, but there was notable variation in the dominant guild at each site. When grouped by habitat, wood saprotrophs were also the most abundant guild (including in the grasslands of the Pacific Northwest region), with the next most abundant guilds in conifer‐dominated forests, oak‐dominated forests, and grasslands being ectomycorrhizal, litter saprotrophs, and soil saprotrophs, respectively (Figure S5). Additionally, sporocarp morphology varied considerably within and among guilds, and included gasteroid, gilled, pored, resupinate, secotioid, and other macrofungal growth forms (Table S2).

The 10 genera with the highest relative abundance in each site, calculated using the number of OTUs in that site, varied in composition as well as occupancy across samples (Table 1). Many of these genera were present across multiple sites, including *Armillaria* (found in 5 sites), *Rhizopogon* (4), *Peziza* (3), *Elaphomyces* (2), *Geopyxis* (2), and *Lactarius* (2), but all 8 sites differed in the genus with the highest relative abundance. Several taxa were ranked highly despite occurring in only a few samples, suggesting localised dominance or relatively isolated sporulation events. For example, *Disciseda* was the most abundant genus in the Southeast ecoregion site but was only detected in a single unburned oak sample in that site. In contrast, the same genus was both abundant and temporally widespread in the Southern Rockies ecoregion site, being found in 75% of samples. Interestingly, the genus *Elaphomyces*, which produces hypogeous sporocarps (a.k.a. truffles), was the highest ranked taxon in the Great Lakes ecoregion site, and truffle‐forming genera were present among the 10 most relatively abundant genera in 7 of the 8 sites.

**TABLE 1:** The 10 most abundant genera of aerial macrofungi in each site, ranked in descending order by mean relative abundance.

	DSW	GRTL	NE	PNW	PRAI	SE	SROC	TAIG
Rank 1	*Lepiota* (0.05)	*Elaphomyces* (0.03)	*Bovista* (0.04)	*Crucibulum* (0.01)	*Kuehneromyces* (0.01)	*Disciseda* (0.00)	*Lycoperdon* (0.89)	Alpova (0.25)
Rank 2	*Agaricus* (0.50)	*Peziza* (0.07)	*Baeospora* (0.37)	*Paralepista* (0.03)	*Inonotus* (0.06)	*Rhizopogon* (0.18)	*Disciseda* (0.75)	*Pseudotrichaptum* (0.75)
Rank 3	*Hypholoma* (0.05)	*Gyromitra* (0.01)	*Cortinarius* (0.70)	*Iodophanus* (0.02)	*Gloeostereum* (0.01)	*Armillaria* (0.16)	*Rhizopogon* (0.14)	*Diplomitoporus* (0.75)
Rank 4	*Rhodocollybia* (0.05)	*Armillaria* (0.37)	*Lactarius* (0.15)	*Hymenogaster* (0.01)	*Sclerogaster* (0.01)	*Hymenochaetopsis* (0.33)	*Calonarius* (0.14)	*Paxillus* (0.83)
Rank 5	*Hypoxylon* (0.11)	*Bolbitius* (0.01)	*Hygrophoropsis* (0.19)	*Rhizopogon* (0.21)	*Myxomphalia* (0.01)	*Tuber* (0.01)	*Bovista* (0.79)	*Xerombrophila* (0.17)
Rank 6	*Radulomyces* (0.02)	*Lactarius* (0.01)	*Porodisculus* (0.19)	*Geopyxis* (0.16)	*Phlebiopsis* (0.14)	*Tricholoma* (0.28)	*Entoloma* (0.21)	*Mycena* (0.75)
Rank 7	*Armillaria* (0.20)	*Porodisculus* (0.05)	*Megacollybia* (0.41)	*Hypholoma* (0.77)	*Fomitopsis* (0.02)	*Myxomphalia* (0.00)	*Gautieria* (0.04)	*Coprinellus* (0.83)
Rank 8	*Rhizopogon* (0.20)	*Agrocybe* (0.02)	*Tylopilus* (0.44)	*Peziza* (0.17)	*Pachyphlodes* (0.11)	*Elaphomyces* (0.10)	*Peziza* (0.25)	*Cantharellula* (0.67)
Rank 9	*Coprinus* (0.11)	*Arachnion* (0.01)	*Radulomyces* (0.81)	*Fomitopsis* (0.38)	*Geopyxis* (0.01)	*Peniophorella* (0.72)	*Athelia* (0.04)	*Armillaria* (0.25)
Rank 10	*Hymenochaete* (0.02)	*Tulosesus* (0.01)	*Armillaria* (0.19)	*Pholiota* (0.68)	*Scleroderma* (0.12)	*Melanoleuca* (0.01)	*Boletus* (0.32)	*Stereum* (0.75)

*Note:* The occupancy of each genus (proportion of samples containing that genus) is reported in parentheses below the name of that genus. Many abundant taxa have low occupancy values, implying that spore traps capture stochastic sporulation events that often produce a large number of spores.

## 4 Discussion

### 4.1 Continental Structuring by Site Location

At the broadest spatial scale, our results were consistent with the first hypothesis that site location would be the primary driver of aerial macrofungal community abundance and structure, which aligns well with a recent survey of the global aerial fungal community (Abrego et al. 2024) as well as with other global fungal sampling efforts (Tedersoo et al. 2014). The site‐level differences in both macrofungal community abundance and richness broadly tracked gradients in climate, following well‐established latitudinal patterns of fungal diversity (Tedersoo et al. 2014). Community composition was also primarily structured by site location, although once uneven habitat representation across sites was accounted for, habitat no longer significantly explained any continental‐scale beta diversity trends. This result suggests that there is no single habitat‐defined composition of the aerial macrofungal community across sites, but rather that dispersal limitation and regional species pools may override habitat filtering at broad spatial scales (Talbot et al. 2014). Curiously though, temperature and precipitation varied in their correlation with aerial macrofungal abundance and OTU richness within the eight sites, and subtle effects of sampling year and season of collection emerged. Together, these latter results suggest that local ecological and temporal context plays a large role in shaping finer‐scale patterns of aerial macrofungal community richness and abundance. Additionally, many OTUs were constrained to a single site and/or habitat, leading to high community variation among sites as well as large dissimilarity between samples within the same site and collected within the same sampling window, consistent with some role of stochasticity also being present (Abrego et al. 2024).

### 4.2 Habitat Filtering Between Forests and Grasslands

Across sites, forests and grasslands exhibited clear habitat‐associated differences in aerial macrofungal DNA abundance as well as OTU richness. These patterns may reflect greater substrate availability and favourable microclimatic conditions within forests, combined with strong host specificity and dominance of ectomycorrhizal (ECM) fungi in conifer systems (e.g., *Rhizopogon*, *Suillus*; Molina et al. 1992), contrasted with a greater prevalence of litter‐decaying fungi in oak forests and soil saprotrophs in grasslands (Awad et al. 2019; Iacono et al. 2025). Within sites containing multiple habitats, community composition also significantly diverged, providing support for a combination of dispersal limitation (e.g., 30 km habitat separation at the Desert Southwest ecoregion site) and local‐scale habitat filtering when sampled habitats occurred in close proximity (e.g., the Prairie ecoregion site). The guild patterns reinforce these hypotheses: ECM fungi dominated conifer sites but were functionally ‘replaced’ by litter saprotrophs in oak forests and soil saprotrophs in grasslands, consistent with prominent guild shifts along forest–grassland gradients (Wang, Wang, et al. 2023). Somewhat unexpectedly, wood‐decay fungi remained abundant across all habitats, including grasslands. This likely reflects some combination of cross‐habitat spore dispersal, persistence of woody substrates in open systems, and/or broader ecological flexibility within this guild of fungi (Kipping et al. 2022). Many wood decay fungi, particularly polypores, are known to have prolific spore generation and high spore dispersal capacity, which may explain much of this cross‐habitat signal (Norros et al. 2012). Despite pronounced differences in composition and guild representation, aerial macrofungal abundance and richness within sites remained similar across habitats. These results indicate that, while the habitat identity influences which macrofungal groups dominate aerial pools, they may do so without strongly altering overall load, implying that distinct fungal guilds may achieve comparable reproductive output when locally dominant.

### 4.3 Limited Effects of Burn History

Perhaps the most surprising result was the lack of detectable effect of recent fire on aerial macrofungal DNA abundance and richness. Known pyrophilous taxa were present and among the most abundant genera at several sites (e.g., *Geopyxis* and *Peziza* in the Pacific Northwest ecoregion site; *Coprinus* in the Desert Southwest ecoregion site; *Pholiota* in the Northeast, Pacific Northwest, and Taiga ecoregion sites), yet recently burned plots did not differ significantly from unburned plots in any measures of alpha diversity. We did, however, detect small by significant differences in aerial macrofungal community composition within sites across burn treatments, partially due to some taxa mentioned above as well as *Scleroderma* and *Pachyphlodes* in the Prairie ecoregion site and *Xylobolus* in the Southeast ecoregion site. We suspect the lack of detected alpha diversity trends and limited beta diversity trends are the result of two interacting factors. First, our spore trap sampling took place at least 6 months after the most recent fire. Many fire‐adapted systems (e.g., grasslands and oak forests) recover, or mostly recover, within one to two months (Hopkins et al. 2023; Tredennick et al. 2014), and many pyrophilous taxa are known to fruit and release spores rapidly, often within two weeks post‐fire (Bruns et al. 2020; Castaño et al. 2017). Thus, the period of dominance of pyrophilous fungi in the aerial macrofungal communities likely passed before our sampling, even in systems with more intense burns (such as the fire in the Pacific Northwest ecoregion conifer forest plots). Second, low severity burns are very patchy, with even large burns showing inconsistent patches of severity across the burn (Buonanduci et al. 2023). Because much of the post‐fire fungal community is shaped by aerial inputs (Barbour et al. 2023) and our estimated lower‐bound spore input radius was on the kilometre scale, traps in burned areas likely received substantial inoculum from adjacent unburned patches, masking localised pyrophilous enrichment and reducing our ability to detect fire‐driven differences in aerial macrofungal communities. While the lack of fire history on aerial macrofungal alpha and beta diversity seems most likely due to the delayed timing of our sampling, other studies within our system have seen fire history effects in other ecosystem compartments, such as increased abundance, litter decomposition, and higher sporocarp production by pyrophilous fungi recently after burns in the Pacific Northwest (Burrill et al. 2025).

### 4.4 Limited Effective Dispersal Distance

The patterns of spatial dissimilarity in aerial macrofungal communities across and within sites found in this study were consistent with those observed in soil micro‐ and macro‐fungal assemblages across broad geographic scales (Talbot et al. 2014; Banerjee et al. 2024). As seen in other fungal studies, community similarity declined predictably with geographic distance while remaining locally variable, likely due to fine‐scale habitat filtering and stochastic dispersal (Kivlin et al. 2011; Bahram et al. 2013; Barberán et al. 2015; Yuan et al. 2018). These distance‐decay relationships suggest that even aerially dispersed macrofungal propagules are strongly structured by environmental heterogeneity and dispersal limitation. Species accumulation curves bolster this interpretation: steep initial increases near spore traps before approaching site‐specific plateaus suggest tight spatial clustering where sampling was intensive (e.g., within the Southeast and Great Lakes ecoregion sites), while the absence of clear asymptotes in other sites indicates incomplete sampling of local diversity (e.g., within the Southern Rockies and Desert Southwest ecoregion sites). Low predicted asymptotes from Michaelis–Menten fits reinforce that a large portion of aerial macrofungal diversity remains undetected, consistent with other large‐scale fungal biodiversity surveys (Egidi et al. 2019).

Patterns of taxonomic overlap between DNA‐based aerial macrofungal data and community‐sourced iNaturalist observations mirrored the accumulation dynamics. The proportion of shared taxa declined sharply with distance to the spore traps, with a breakpoint near 3 km marking a conservative spatial limit at which local iNaturalist observations ceased to provide meaningful signal in the macrofungal community captured. Locations with intense long‐term survey efforts (Southeast and Pacific Northwest) showed a higher asymptotic value and consistent overlap while less well‐sampled locations like the Desert Southwest or Prairie exhibited low asymptote values and rapid initial slopes, suggesting under sampling of inconspicuous or short‐lived fruiting bodies (Figure S3). Differences in overlap ratios among sites also highlight how habitat structure and survey context shape perceived macrofungal diversity. Specifically, sites with high proportions of taxa found in both the spore traps and community data, such as the Taiga or Desert Southwest ecoregion sites, likely represent areas of high local sporulation and limited observer activity, whereas those dominated by taxa seen only in iNaturalist, such as the Pacific Northwest and Southern Rockies ecoregion sites, suggest strong long‐distance dispersal potential coupled with dense observer networks in heterogeneous landscapes (Geurts et al. 2023; Su et al. 2022). Together, the combined behaviour of the accumulation curves and observational taxa overlap indicate that perceived macrofungal diversity may be strongly shaped by both spatial scale and sampling intensity, and that integrating molecular and community‐sourced data provides a more holistic view of macrofungal diversity and dispersal by bridging the gap between ecological sampling and public biodiversity monitoring.

### 4.5 Caveats and Future Research

While our study reveals both local‐ and continental‐scale patterns in aerial macrofungal communities, we acknowledge multiple limitations and highlight opportunities for future research. First, local variation was extremely high: abundance, richness, and composition differed even across distances less than a kilometre, indicating that patterns observed here should not be universally extrapolated within ecoregions reported. Although ecoregional designations capture broad climatic and vegetative similarities, macrofungal dispersal and assembly appear to operate at finer spatial scales, suggesting that predictive models would benefit from incorporating microsite conditions, landscape structure, and atmospheric drivers such as wind direction and speed (Pouska et al. 2016; Grinn‐Gofroń et al. 2020). Expanding spatial gradients, particularly into currently under sampled portions of North America (e.g., Mexico, the Caribbean), will also improve the generality of continental‐scale inference (Guzmán 1998). Second, the habitats in this study were sampled discretely rather than across continuous gradients. As a result, we could detect differences among forest and grassland habitats but were unable to quantify the rate or spatial extent of community transitions across ecotones, which has been shown to be ecologically meaningful in other studies (Ma et al. 2017; Wang, Smith, et al. 2023). In addition, the partial factorial design, especially for fire history, reduced our statistical power in some regions (e.g., the Northeast ecoregion site). Future sampling that systematically spans habitat edges and gradients, burn severities, and post‐fire recovery time would help distinguish deterministic shifts from stochastic variation in spore inputs. Third, within‐site heterogeneity was high, and single spore traps per subplot occasionally reflected outsized contributions from intense, short‐duration sporulation events. While similar trap designs have been successful in previous studies (Peay et al. 2012; Borgmann‐Winter et al. 2023), we suggest that the use of multiple types of trap designs (Schlegel et al. 2024), or a higher density of traps (such as greater than three traps within a square‐meter area), may better capture airborne community structure when targeting whole community inference (Naranjo‐Orrico et al. 2025). Finally, pairing aerial DNA surveys with iNaturalist records provides a powerful bridge between observer‐based and molecular biodiversity data, but each source has inherent biases. For fungi, iNaturalist records are uneven across regions, skew toward conspicuous Basidiomycota, and underrepresent inconspicuous macrofungi (e.g., crusts, rusts, small agarics) unless specifically targeted. Conversely, DNA‐based data captures cryptic taxa but includes many undescribed or unverified lineages, introducing uncertainty in taxonomic resolution. As such, the continued development of curated reference libraries and integrative sampling approaches will improve the ecological interpretability of aerial fungal datasets. Although our design was not optimised to quantify the full spatial continuum of habitat and disturbance effects, our findings do emphasise that aerial macrofungal communities respond to both broad ecological gradients and highly localised processes. Recognizing this multiscale and nested structure will be critical for advancing predictive models of fungal dispersal, community assembly, and ecosystem function.

## Author Contributions

B.A.R., A.E.A., J.C., J.M.D., P.G.K., D.J.L., M.E.S., A.W.W., and J.U. conceived of the study and secured funding. These authors and H.M.B., M.V.C., H.A.D., J.J.L., J.W., C.B.W. collected data for the study. A.W.R. and E.K.R. conducted all of the bioinformatic and statistical analyses, with guidance from P.G.K. A.W.R. and P.G.K. co‐wrote the paper, with all authors providing edits and approving the final version.

## Funding

Funding was provided by a National Science Foundation (NSF) Macrosystems Biology and NEON‐Enabled Science Grant DEB‐2106130, with research also facilitated by multiple NSF LTER Grants (CDR: DEB‐1831944, HJA: DEB‐2025755, NIWOT: DEB‐1637686 and DEB‐2224439, DEB‐2425352, DEB‐0620652, DEB‐1234162 and DEB‐1831944).

## Conflicts of Interest

The authors declare no conflicts of interest.

## Supporting information


**Table S1:** Information about the identity, location, and general climate and ecosystem conditions of each site and treatment used for the study. Abbreviated versions of each site are found in parentheses for ease of reference.

**Table S2:** Top 10 aerial macrofungal genera by mean rank abundance for each site, calculated using the number of OTUs found in that ecoregion. Below the name is the common growth form in parentheses.

**Table S3:** List of taxa designated as macrofungi for this study. Included and excluded taxa are based consultation of primary literature and curation by Bitty Roy, Matt Smith, as well as other collaborators within the CLIMUSH project.

**Table S4:** Table denoting the climatic and temporal trends within sites based on mixed linear effect models with subplot as a random variable due to repeated testing for both macrofungal DNA abundance and macrofungal OTU richness. Non‐significant trends are reported as “ns” within the cell. Significant trends in MMT or MMP are denoted with a “+” for positive trends and a “−” for negative trends. Season and year are reported as the category that is higher (2022 or 2023 and “E” for early season and “L” for late season).

**Figure S1:** mec70366‐sup‐0005‐Supinfo.docx. Within‐site effects of habitat and burn history on adjusted aerial macrofungal abundance and richness. Habitat (left) had a significant effect on abundance (a) and OTU richness (c) only in the Pacific Northwest ecoregion site (all *p*‐values < 0.001). Burn history had no significant effect in qPCR abundance (b) nor OTU richness (d) regardless of site identity.
**Figure S2:** Composition of aerial macrofungal communities by habitat for each site. Centroids in NMDS plots are denoted by large dots, dispersion denoted by faint fill. Habitats with significant differences in centroid position are marked with an asterisk (*p* < 0.001) and the stress of the model is reported for all graphs.
**Figure S3:** Sporocarp species accumulation curves by site. Sporocarp data were collected both from the research team and members of the iNaturalist community. The species curve is shown in black and the Michaelis–Menten Model curve with error is shown in blue. The ‘a’ statistic denotes the species saturation of the model, the number of expected species given an infinite sampling range i.e., the asymptote, and ‘b’ denotes the distance from the spore trap where half of the predicted maximum species have been observed. All sites are combined and averaged in panel a, with each separate site being fit with a separate model in panel b.
**Figure S4:** Proportional breakpoint models by site. Black lines represent a non‐linear least squares model of the proportion of genera observed in iNaturalist sporocarp data that were also captured in spore traps within that site. Proportions are graphed against distance of that observation from the nearest spore trap. The dotted line and reported number show a shift in slope as determined using a breakpoint model, indicating where sporocarp observations are no longer represented by spore collection.
**Figure S5:** Aerial macrofungal guild distribution by site and habitat. Exact percentages are not reported but note the common switching of ectomycorrhizal taxa (red) in conifer plots for soil saprotrophs (green) in grass plots and letter saprotrophs (blue) in oak plots. This is especially notable in the NE ecoregion and the DSW ecoregion sites.
**Figure S6:** Phenology plots of *Pezizales* (top) and *Agaricomycetes* (bottom) for each sampling site. Data represents number of observations of taxa belonging to those groups retrieved from iNaturalist within the sampling location in years leading up to the spore trap sampling. Black lines represent observations at the location, while light blue lines represent the observations across the county of the sampling point.
**Figure S7:** Histogram of raw read counts within CLIMUSH samples before rarefaction.

## Data Availability

Raw sequencing FASTQ files, along with the associated metadata were deposited in the SRA (BioProject PRJNA1291903). All sporocarp information pulled from iNaturalist can be found at https://www.inaturalist.org and curated observations can be found in the Supporting Information. Processed data files and code used to produce all analyses and figures can be found here (DOI: 10.5281/zenodo.18894239).
