# Supplementary material for: Sporecasting Biogeography Across Scales: Continental Patterns and Local Constraints on Macrofungal Dispersal
Source: Mol Ecol. 2026 May 6;35:e70366. doi: 10.1111/mec.70366 (PMC13147313; doi:10.1111/mec.70366)
Supplement: Supplementary file 5 — Figure S1: mec70366‐sup‐0005‐Supinfo.docx. Within‐site effects of habitat and burn history on adjusted aerial macrofungal abundance and richness. Habitat (left) had a significant effect on abundance (a) and OTU richness (c) only in the Pacific Northwest ecoregion site (all p‐values < 0.001). Burn history had no significant effect in qPCR abundance (b) nor OTU richness (d) regardless of site identity. Figure S2: Composition of aerial macrofungal communities by habitat for each site. Centroids in NMDS plots are denoted by large dots, dispersion denoted by faint fill. Habitats with significant differences in centroid position are marked with an asterisk (p < 0.001) and the stress of the model is reported for all graphs. Figure S3: Sporocarp species accumulation curves by site. Sporocarp data were collected both from the research team and members of the iNaturalist community. The species curve is shown in black and the Michaelis–Menten Model curve with error is shown in blue. The ‘a’ statistic denotes the species saturation of the model, the number of expected species given an infinite sampling range i.e., the asymptote, and ‘b’ denotes the distance from the spore trap where half of the predicted maximum species have been observed. All sites are combined and averaged in panel a, with each separate site being fit with a separate model in panel b. Figure S4: Proportional breakpoint models by site. Black lines represent a non‐linear least squares model of the proportion of genera observed in iNaturalist sporocarp data that were also captured in spore traps within that site. Proportions are graphed against distance of that observation from the nearest spore trap. The dotted line and reported number show a shift in slope as determined using a breakpoint model, indicating where sporocarp observations are no longer represented by spore collection. Figure S5: Aerial macrofungal guild distribution by site and habitat. Exact percentages are not reported but note the common switch [file MEC-35-e70366-s002.docx]

# **Supplementary Information**

Quantitative PCR conditions and methods

For the qPCR reactions, serial dilutions (undiluted, 1:8, 1:64) of DNA from each sample were amplified with the ITS1F-ITS2 primer pair (White et al., 1990; Gardes & Bruns, 1993) in a 6 μL reaction volume using KAPA HiFi polymerase on a Qiagen QS5 thermocycler. Amplification conditions consisted of 5 min at 95 °C, followed by 25 cycles of 20 s at 98 °C, 15 s at 55 °C, and then 1:00 min at 72 °C. Quantifications were based on C_t_ values compared to fungal standard curves and expressed as the log10 number of ITS copies per filter.

Clustering ASVs into OTUs

In order to cluster the assigned ASVs into OTUs, a custom pipeline developed by Luis Morgado and then updated and run by Eivind Ronold (). First the ITS1 region of each ASV was extracted using ITSx (Bengtsson-Palme et al. 2013). Leaving the highly conserved ITS1 regions on each ASV may inflate the similarity between ASVs which would adjust the clustering of later steps. We then used VSEARCH (Rognes et al. 2016) to cluster these sequences at a 97% similarity threshold. A de novo chimera-check and removal step was also included within VSEARCH to eliminate potentially chimeric sequences that have been shown to be missed by DADA2 (Pauvert et al, 2018).

(Table Submitted as CLIMUSH_SuppTable_1.xlsx)

**Supplementary Table 1 -** Information about the identity, location, and general climate and ecosystem conditions of each site and treatment used for the study. Abbreviated versions of each site are found in parentheses for ease of reference.

Information about each site was collected from the site webpage and/or in personal communication with site managers: Roger Reuss & Jamie Hollingsworth (BNZ), Jason Blazar & Ed Alverson (Buford Recreation Area), Forest Isbel (CDR), Audrey Barker-Plotkin (Harvard Forest), Mark Schultz (HJA), John Blair (KON), Nancy Emory (NIW), Andrew Rappe (ORD), Brett Blume & Mitchell McClaren (SRE). Locations and Elevation of each location were taken at subplot S5 (see Supplementary Figure 1). Temperature and precipitation were 30-year averages (1990-2020) for all sites except for Bonanza Creek, and were exported from the PRISM Group (https://prism.oregonstate.edu, data created 4 Feb 2014, accessed 16 Dec 2020). All climatic readings not taken from PRISM were collected from a nearby airport.

**
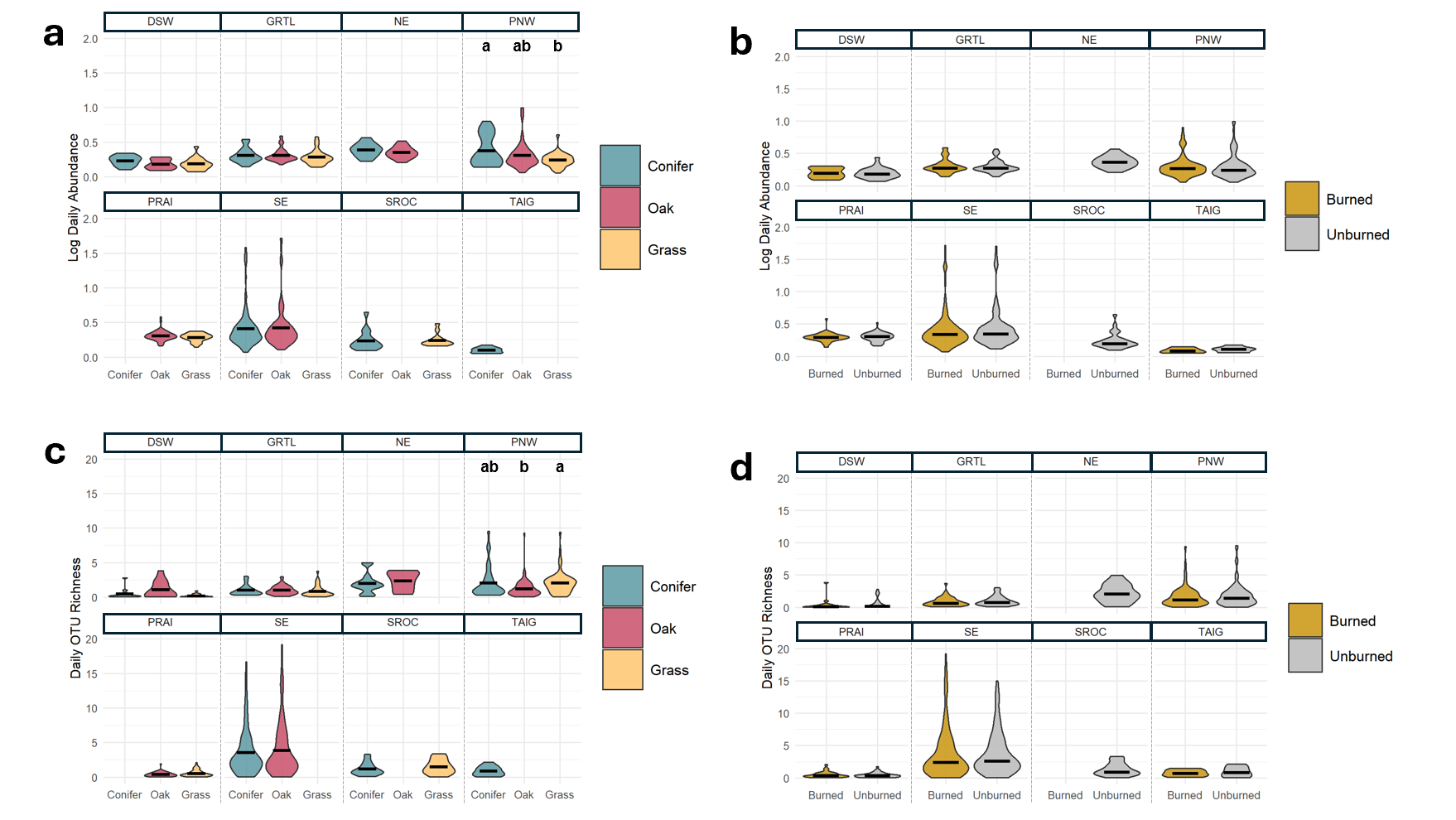
**

**Supplementary Figure 1.** Within-site effects of habitat and burn history on adjusted aerial macrofungal abundance and richness. Habitat (left) had a significant effect on abundance (a) and OTU richness (c) only in the Pacific Northwest ecoregion site (all p-values < 0.001). Burn history had no significant effect in qPCR abundance (b) nor OTU richness (d) regardless of site identity.


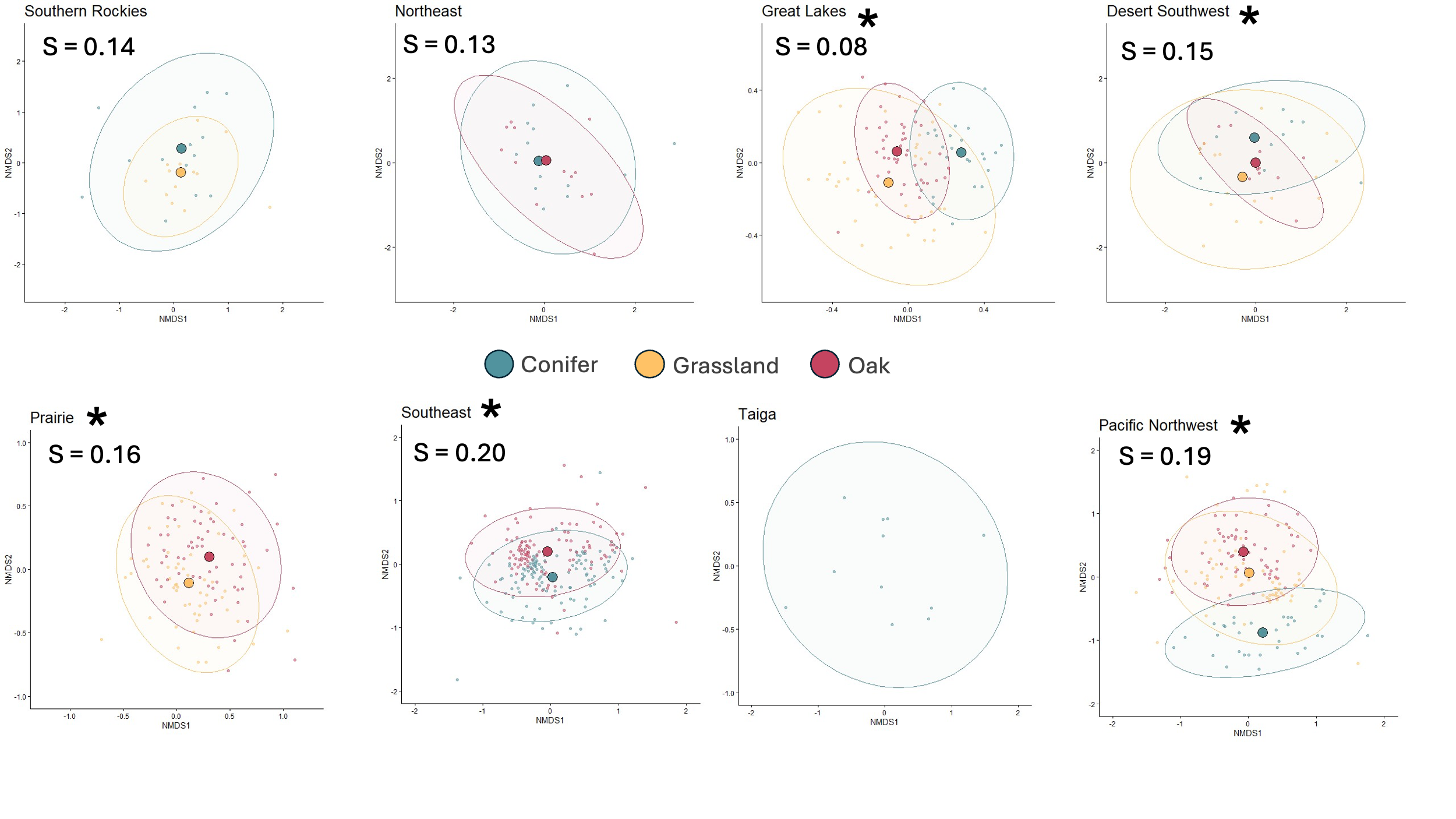


**Supplementary Figure 2.** Composition of aerial macrofungal communities by habitat for each site. Centroids in NMDS plots are denoted by large dots, dispersion denoted by faint fill. Habitats with significant differences in centroid position are marked with an asterisk (p < 0.001) and the stress of the model is reported for all graphs.

**
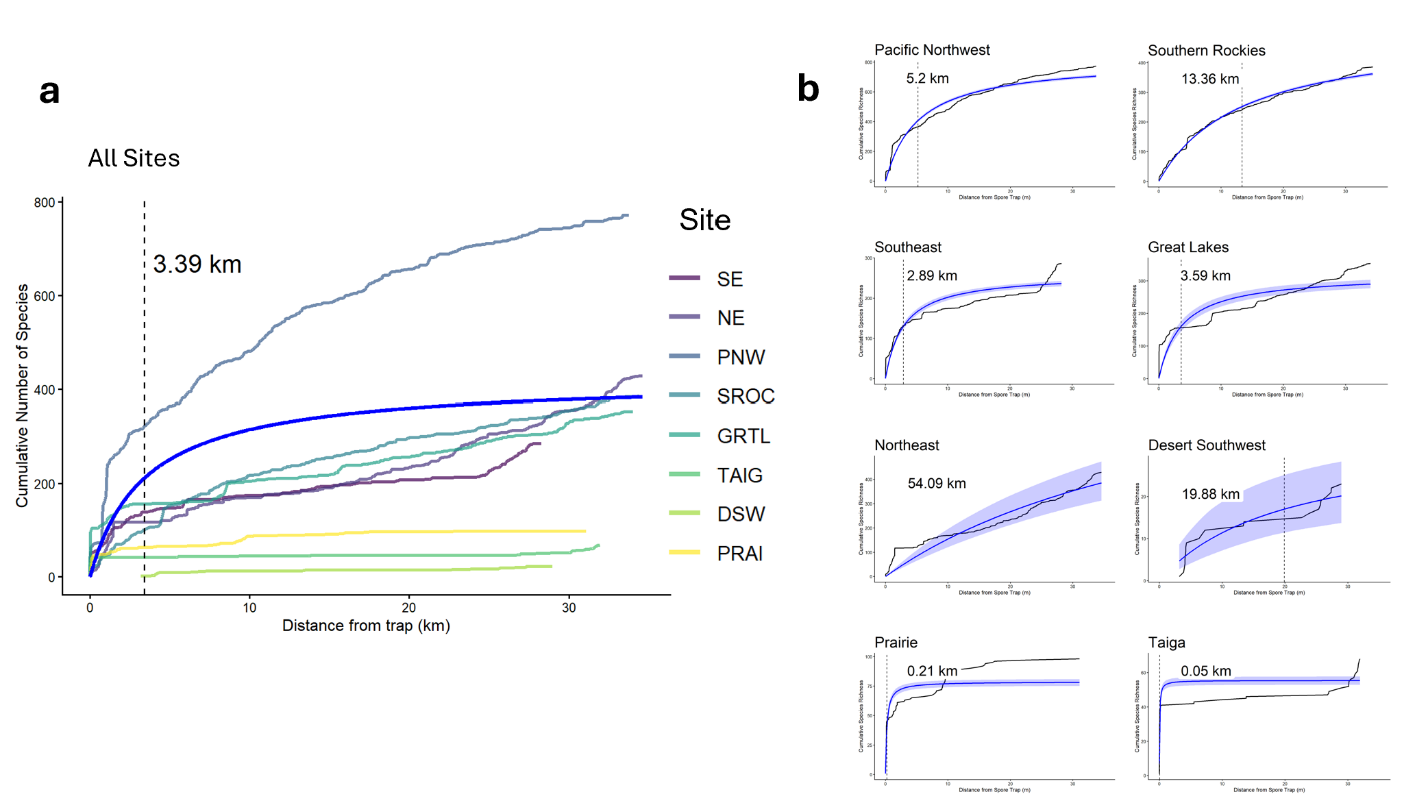
**

**Supplementary Figure 3.** Sporocarp species accumulation curves by site. Sporocarp data were collected both from the research team and members of the iNaturalist community. The species curve is shown in black and the Michaelis-Menten Model curve with error is shown in blue. The ‘a’ statistic denotes the species saturation of the model, the number of expected species given an infinite sampling range i.e. the asymptote, and ‘b’ denotes the distance from the spore trap where half of the predicted maximum species have been observed. All sites are combined and averaged in panel **a**, with each separate site being fit with a separate model in panel **b.**

**
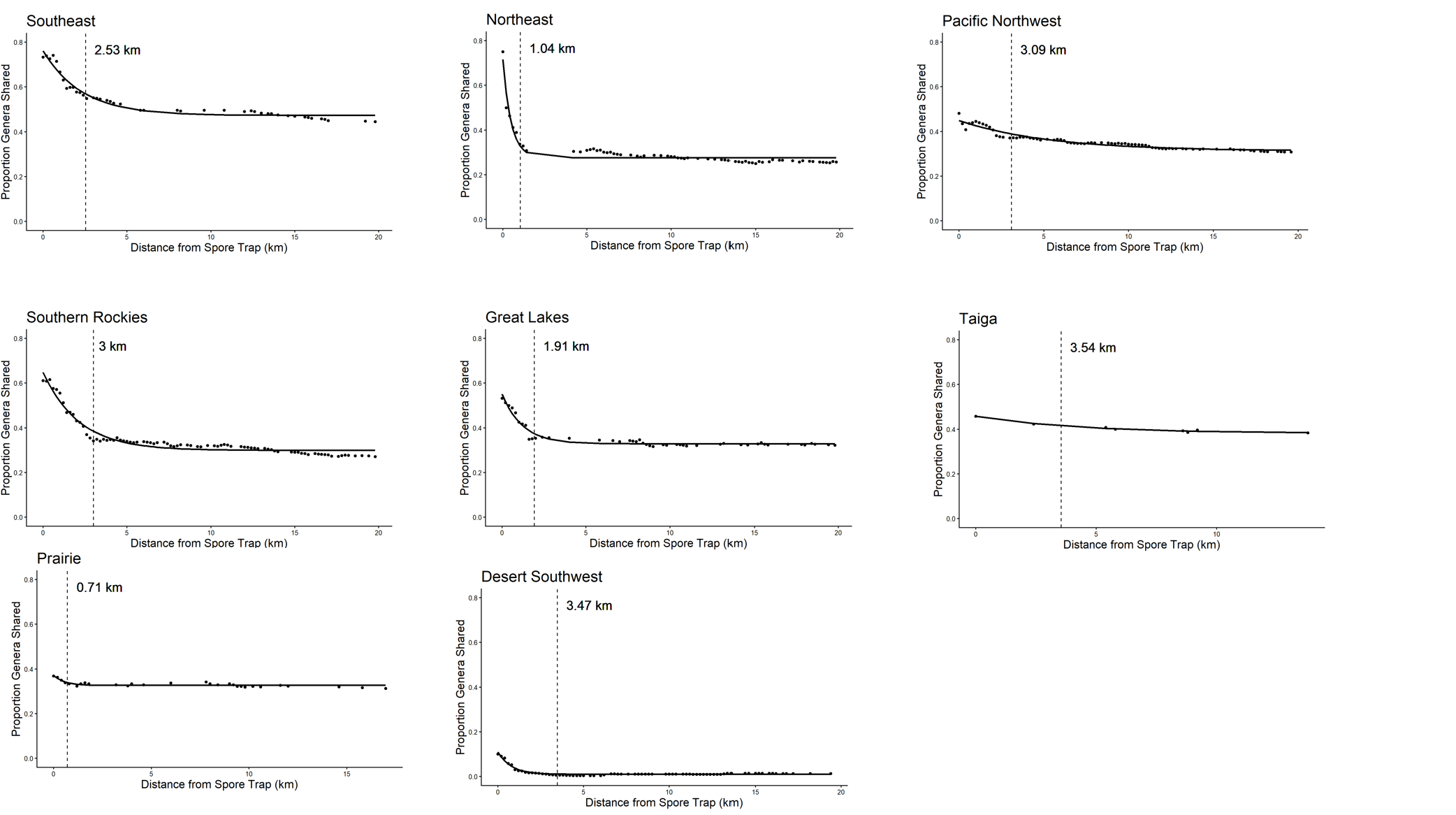
**

**Supplementary Figure 4.** Proportional breakpoint models by site. Black lines represent a non-linear least squares model of the proportion of genera observed in iNaturalist sporocarp data that were also captured in spore traps within that site. Proportions are graphed against distance of that observation from the nearest spore trap. The dotted line and reported number show a shift in slope as determined using a breakpoint model, indicating where sporocarp observations are no longer represented by spore collection.

**
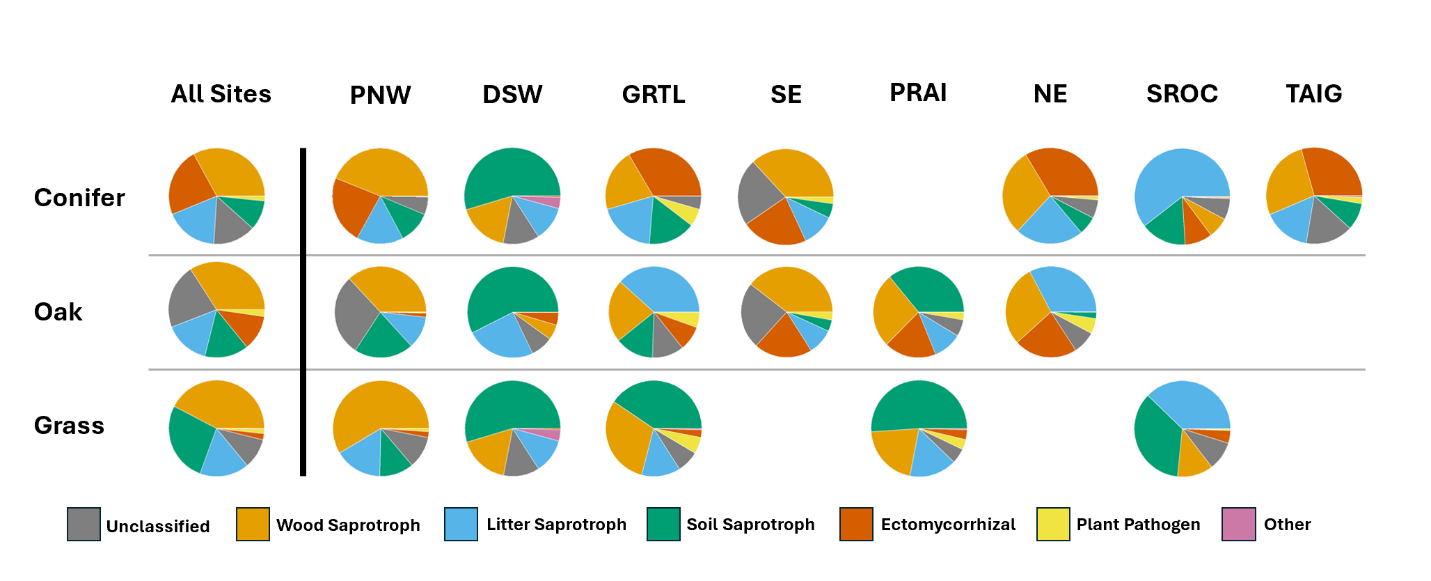
**

**Supplementary Figure 5.** Aerial macrofungal guild distribution by site and habitat. Exact percentages are not reported but note the common switching of ectomycorrhizal taxa (red) in conifer plots for soil saprotrophs (green) in grass plots and letter saprotrophs (blue) in oak plots. This is especially notable in the NE ecoregion and the DSW ecoregion sites.

Spore Trap Collection Timeframes

In the first year, spore traps were deployed continuously during periods of regular precipitation at all sites except the Harvard Forest, MA in the Northeast ecoregion. In the second year, deployment was targeted in spring and fall to coincide with peak fruiting periods of Ascomycota and Basidiomycota sporocarps. The specific periods within each site can be found in figure 1 subpanel c. These seasonal peaks were identified from site-specific phenology curves constructed using iNaturalist observation data nearby to the sites in years prior to sampling based on the orders of *Pezizales* as a representative of Ascomycota and *Agaricomycetes* as a representative of the Basidiomycota. Specific phenology curves for each site can be found below.


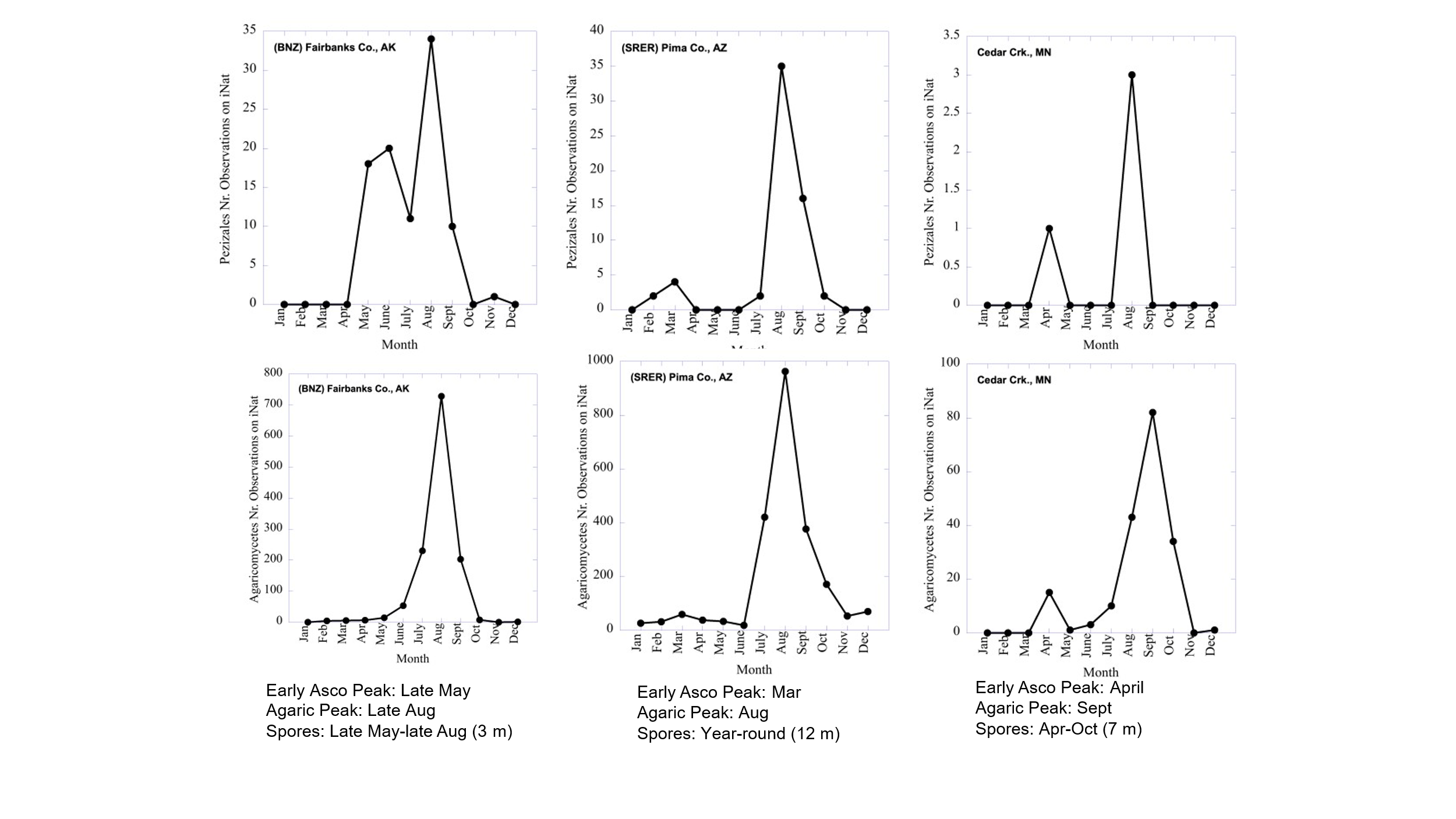


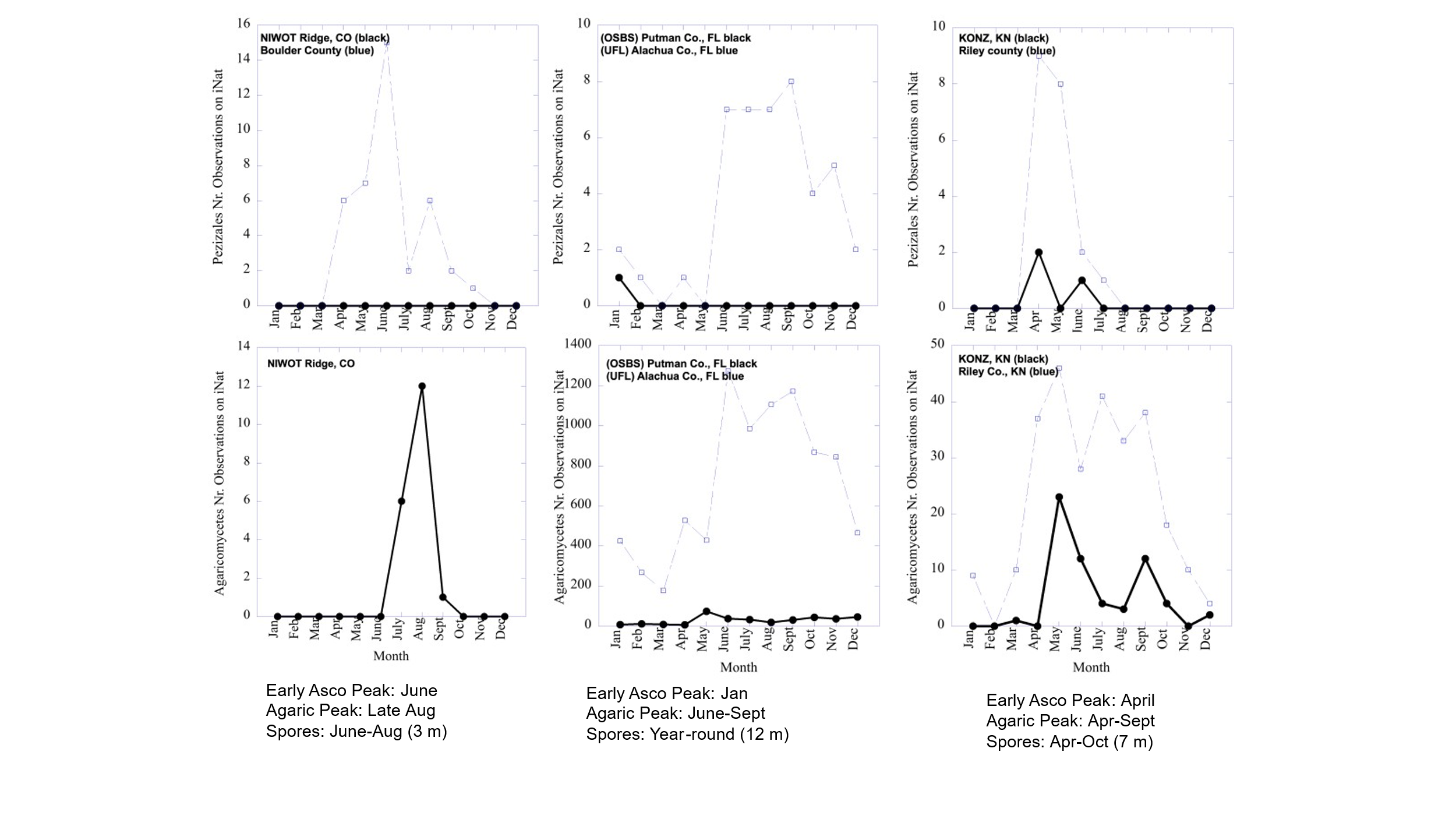


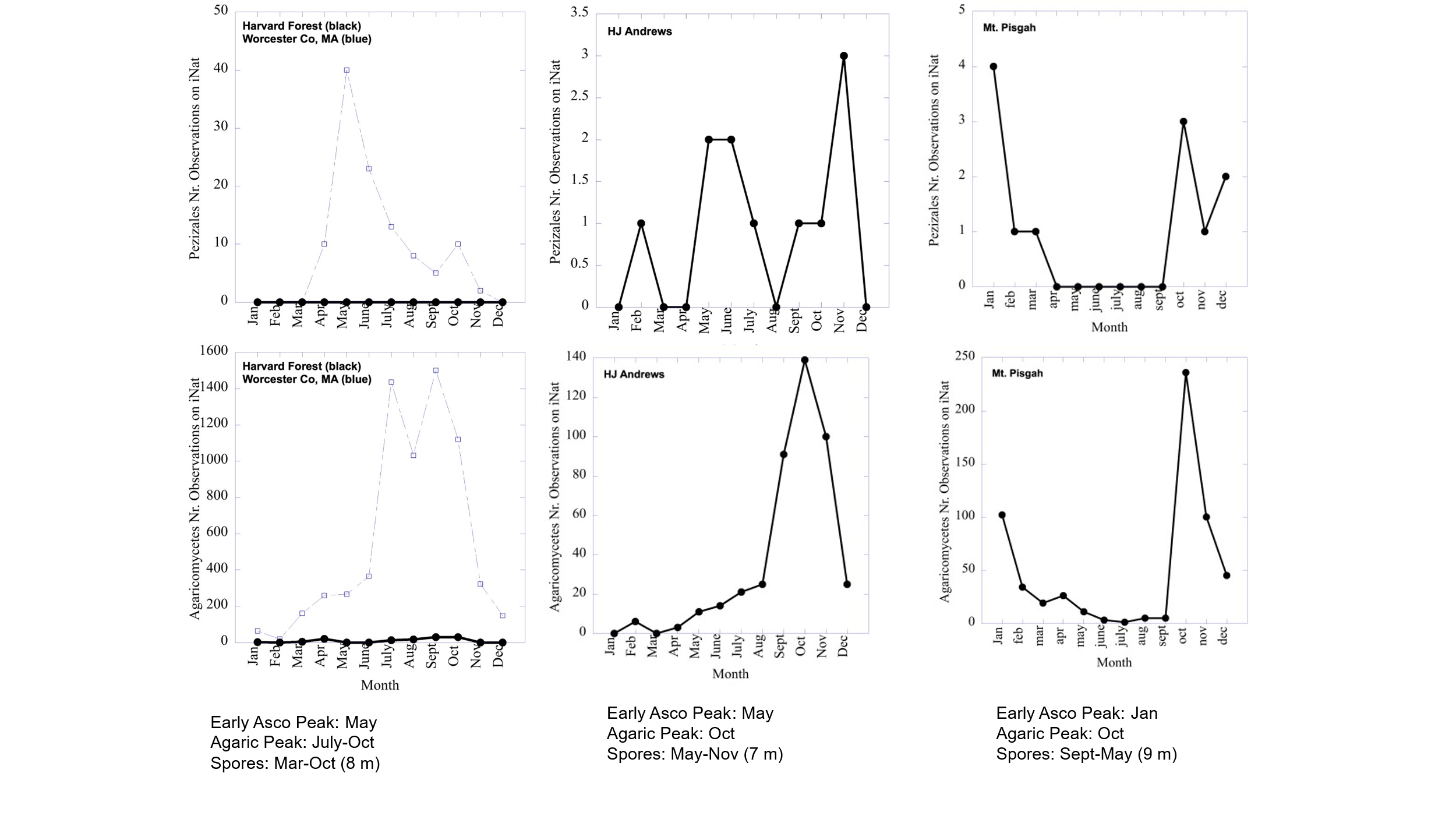


**Supplementary Figure 6.** Phenology plots of *Pezizales* (top) and *Agaricomycetes* (bottom) for each sampling site. Data represents number of observations of taxa belonging to those groups retrieved from iNaturalist within the sampling location in years leading up to the spore trap sampling. Black lines represent observations at the location, while light blue lines represent the observations across the county of the sampling point.


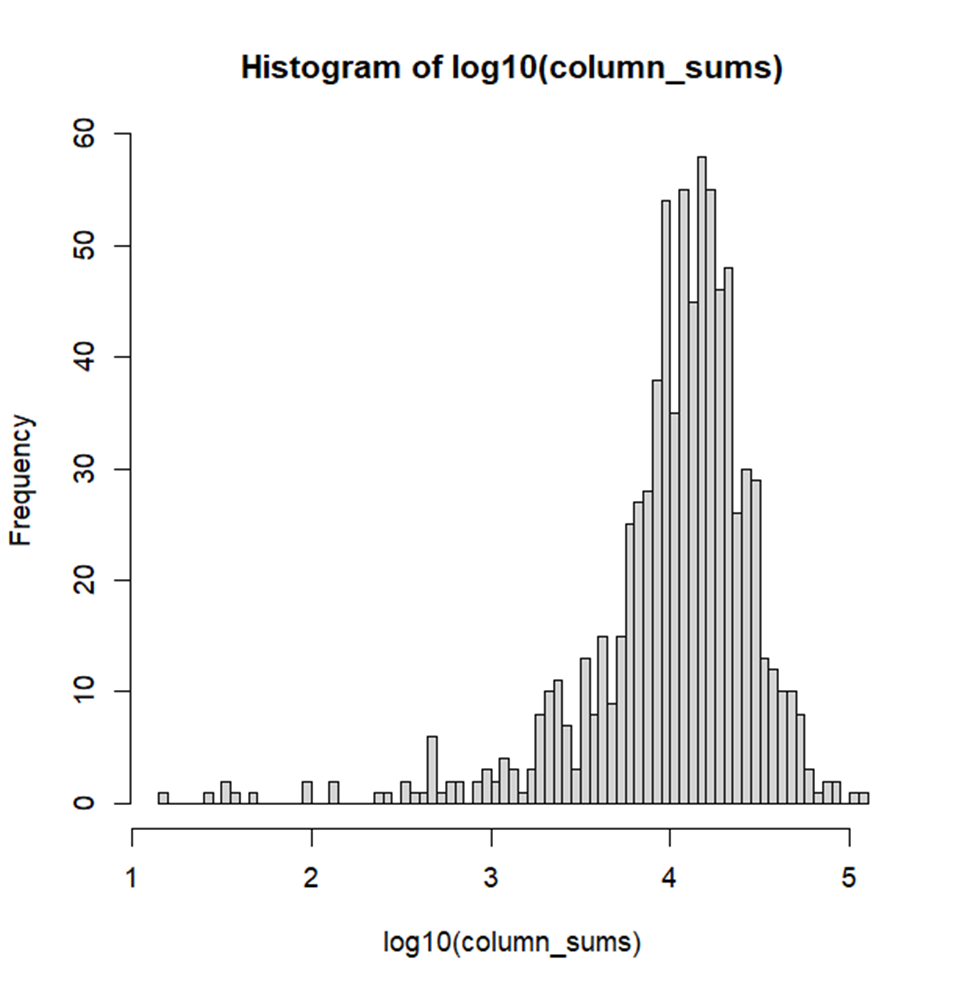


**Supplementary Figure 7.** Histogram of raw read counts within CLIMUSH samples before rarefaction.

(Submitted as CLIMUSH_SuppTable_2.xlsx)

**Supplementary Table 2.** Top 10 aerial macrofungal genera by mean rank abundance for each site, calculated using the number of OTUs found in that ecoregion. Below the name is the common growth form in parentheses.

(Submitted as CLIMUSH_SuppTable_3.xlsx)

**Supplementary Table 3.** List of taxa designated as macrofungi for this study. Included and excluded taxa are based on Mueller G.M. et al., (2007), Thiers & Halling 2018, and expert curation by Bitty Roy, Matt Smith, as well as other collaborators within the CLIMUSH project.

(Submitted as CLIMUSH_SuppTable_4.xlsx)

**Supplementary Table 4.** Table denoting the climatic and temporal trends within sites based on mixed linear effect models with subplot as a random variable due to repeated testing for both macrofungal DNA abundance and macrofungal OTU richness. Non-significant trends are reported as “ns” within the cell. Significant trends in MMT or MMP are denoted with a “+” for positive trends and a “-“ for negative trends. Season and year are reported as the category that is higher (2022 or 2023 and “E” for early season and “L” for late season).

**References**:

Bengtsson-Palme J, Ryberg M, Martmann M et al (2013) Improved software detection and extraction of ITS1 and ITS2 from ribosomal ITS sequences of fungi and other eukaryotes for analysis of environmental sequencing data. Methods Ecol Evol, 4:9 14-919. https://doi.org/10.1111/2041-210X.12073

Gardes M, Bruns TC, (1993) ITS primers with enhanced specificity for basidiomycetes - application to the identification of mycorrhizae and rusts. Molecular Ecology 2:113-118. https://doi.org/10.1111/j.1365-294X.1993.tb00005.x

Pauvert C, Buée M, Laval V et al (2019) Bioinformatics matters: The accuracy of plant and soil fungal community data is highly dependent on the metabarcoding pipeline. Fungal Ecology 41:22-33. https://doi.org/10.1016/j.funeco.2019.03.005

Rognes T, Flouri T, Nichols B, et al (2016) VSEARCH: a versatile open source tool for metagenomics. PeerJ 4:e2584. https://doi.org/10.7717/peerj.2584

White TJ, Bruns TD, Lee SB, et al (1990) Amplification and Direct Sequencing of Fungal Ribosomal RNA Genes for Phylogenetics
